# Sequence-based GWAS meta-analyses for beef production traits

**DOI:** 10.1186/s12711-023-00848-5

**Published:** 2023-10-12

**Authors:** Marie-Pierre Sanchez, Thierry Tribout, Naveen K. Kadri, Praveen K. Chitneedi, Steffen Maak, Chris Hozé, Mekki Boussaha, Pascal Croiseau, Romain Philippe, Mirjam Spengeler, Christa Kühn, Yining Wang, Changxi Li, Graham Plastow, Hubert Pausch, Didier Boichard

**Affiliations:** 1 Université Paris-Saclay, INRAE, AgroParisTech, GABI, 78350 Jouy-en-Josas, France; 2 Animal Genomics, ETH Zurich, 8092 Zurich, Switzerland; 3 Research Institute for Farm Animal Biology (FBN), 18196 Dummerstorf, Germany; 4 Eliance, 75595 Paris, France; 5 INRAE, USC1061 GAMAA, Université de Limoges, 87060 Limoges, France; 6 QualitasAG, 6300 Zug, Switzerland; 7 Agricultural and Environmental faculty, University Rostock, 18059 Rostock, Germany; 8 Present Address: Friedrich-Loeffler-Institut (FLI), 17493 Greifswald, Insel Riems Germany; 9 Lacombe Research and Development Centre, Agriculture and Agri-Food Canada, Lacombe, AB T4L 1W1 Canada; 10 Department of Agricultural, Food and Nutritional Science, Livestock Gentec, University of Alberta, Edmonton, AB T6G 2HI Canada

## Abstract

**Background:**

Combining the results of within-population genome-wide association studies (GWAS) based on whole-genome sequences into a single meta-analysis (MA) is an accurate and powerful method for identifying variants associated with complex traits. As part of the H2020 BovReg project, we performed sequence-level MA for beef production traits. Five partners from France, Switzerland, Germany, and Canada contributed summary statistics from sequence-based GWAS conducted with 54,782 animals from 15 purebred or crossbred populations. We combined the summary statistics for four growth, nine morphology, and 15 carcass traits into 16 MA, using both fixed effects and z-score methods.

**Results:**

The fixed-effects method was generally more informative to provide indication on potentially causal variants, although we combined substantially different traits in each MA. In comparison with within-population GWAS, this approach highlighted (i) a larger number of quantitative trait loci (QTL), (ii) QTL more frequently located in genomic regions known for their effects on growth and meat/carcass traits, (iii) a smaller number of genomic variants within the QTL, and (iv) candidate variants that were more frequently located in genes. MA pinpointed variants in genes, including *MSTN*, *LCORL*, and *PLAG1* that have been previously associated with morphology and carcass traits. We also identified dozens of other variants located in genes associated with growth and carcass traits, or with a function that may be related to meat production (e.g., *HS6ST1*, *HERC2*, *WDR75*, *COL3A1*, *SLIT2*, *MED28*, and *ANKAR*). Some of these variants overlapped with expression or splicing QTL reported in the cattle Genotype-Tissue Expression atlas (CattleGTEx) and could therefore regulate gene expression.

**Conclusions:**

By identifying candidate genes and potential causal variants associated with beef production traits in cattle, MA demonstrates great potential for investigating the biological mechanisms underlying these traits. As a complement to within-population GWAS, this approach can provide deeper insights into the genetic architecture of complex traits in beef cattle.

**Supplementary Information:**

The online version contains supplementary material available at 10.1186/s12711-023-00848-5.

## Background

Beef accounts for approximately 21% of global meat consumption, making it the third most consumed meat in the world after poultry and pork [1]. As such, beef production is a crucial component of the global food system and holds significant economic and cultural importance in many countries. However, it also has a significant environmental footprint [2], which is increasingly considered critical given that, globally, meat production continues to grow [1]. In addition, there is an increasing tendency of breeding dairy cows with beef sires to increase the resource efficiency of the cattle sector [3]. Therefore, it is essential that new technologies, breeding programs (beef on dairy), and practices are developed to make beef production more sustainable and resource-efficient to reduce its environmental impact while avoiding potential negative effects on animal welfare, e.g., via calving difficulties. A first step towards this goal is an improved understanding of the genetic basis of growth and beef traits, which could lead to biology-informed selection taking the potential physiological background of divergent growth into account. By enabling faster and more efficient growth, this approach could reduce the amount of feed, water, and other inputs needed, thereby minimizing the environmental footprint of beef production. Through studying the genetics of beef traits, it is possible to develop breeding programs that optimize beef production in pure- and cross-breeding programs, improve product quality, and reduce its environmental impact, making beef production more sustainable in the long run.

Genome-wide association studies (GWAS) are a powerful tool for identifying genes and genetic variants associated with complex traits [4]. GWAS in cattle are routinely performed at the whole-genome-sequence (WGS) level, thanks to the availability of large reference populations such as that established by the 1000 Bull Genomes Project [5, 6]. Sequence-based association testing enables the identification of candidate causal variants and genes involved in the genetic determinism of complex traits, but the presence of long-range linkage disequilibrium (LD) in cattle can make the differentiation between true causal variants and markers in LD challenging. One means of addressing this problem is the use of meta-analysis (MA) of WGS GWAS results from different populations and breeds, which can be a powerful and accurate way to identify causal variants for complex traits [5, 7–9].

One of the objectives of the H2020 BovReg project is to search for genomic regions that affect various dairy and beef cattle traits using MA at the sequence level. For beef production, five partners from France (INRAE/ULIM), Switzerland (ETH), Germany (FBN), and Canada (UAL) contributed 54,782 animals from 15 populations, both purebred (representing Charolais, Montbéliarde, Normande, Limousine, Blonde d’Aquitaine, Brown Swiss, and Original Braunvieh) and crossbred (Charolais × Holstein, and Angus/Charolais/beef composite). Each partner conducted sequence-based within-population GWAS for four growth, nine morphology, and/or 15 carcass traits. Here, we combined these GWAS results to conduct 16 MA with fixed-effects and z-score methods and performed post-GWAS analyses to identify candidate causal genes and variants.

## Methods

### Ethics statement

All analyses were performed using data from routine recording and genotyping of commercial and research herds of cattle in different European countries. We did not perform any experiments on animals and no ethical approval was required. Data on the Canadian animals were collected in previous projects and all the animals were cared for according to the guidelines established by local Council on Animal Care and approved by local animal user committees.

### Animals and traits

Five partners from France (INRAE and INRAE/ULIM), Switzerland (ETH), Germany (FBN), and Canada (UAL) contributed GWAS results to the present study, obtained from 54,782 animals from 15 populations. The animals were steers, cows, or bulls (19,656 females and 35,126 males), and were analyzed for growth, live morphology, or carcass traits (Table 1). They originated from purebred—i.e., Charolais, Montbéliarde, Normande, Limousine, Blonde d’Aquitaine, Brown Swiss, and Original Braunvieh—or crossbred populations, i.e., Holstein × Charolais and Angus, Charolais, and beef composite. The number of animals per population ranged from 939 to 20,185.

**Table 1:** Features of the populations from the different partners

Partner (country)	Population	Animals	Abbreviation	Number of animals	Group of traits	Phenotype	Number of traits
INRAE (FR)	Charolais	Cows	INRAE_CHA_cows	7999	Growth	YD	2
INRAE (FR)	Charolais	Cows	INRAE_CHA_cows	8501	Live morphology	YD	3
INRAE (FR)	Montbéliarde	Bulls	INRAE_MON_bulls	3226	Live morphology	DYD	2
INRAE (FR)	Normande	Bulls	INRAE_NOR_bulls	2749	Live morphology	DYD	3
INRAE (FR)	Charolais	Steers	INRAE_CHA_steers	4354	Carcass	YD	3
INRAE (FR)	Montbéliarde	Steers	INRAE_MON_steers	4163	Carcass	YD	3
INRAE (FR)	Normande	Steers	INRAE_NOR_steers	2730	Carcass	YD	3
ULIM-INRAE (FR)	Charolais	Steers	INRAE-ULIM_CHA	1059	Growth and carcass	YD	15
ULIM-INRAE (FR)	Limousine	Steers	INRAE-ULIM_LIM	1209	Growth and carcass	YD	15
ULIM-INRAE (FR)	Blonde d’Aquitaine	Steers	INRAE-ULIM_BLA	939	Growth and carcass	YD	15
ETH (CH)	Brown Swiss and Original Braunvieh	Bulls	ETH_bulls	10,419	Carcass	DRP	6
ETH (CH)	Brown Swiss and Original Braunvieh	Cows	ETH_cows	20,185	Carcass	DRP	6
FBN (DE)	Holstein × Charolais F2	Bulls	FBN_bulls	1043	Growth and carcass	YD	13
FBN (DE)	Holstein × Charolais F2	Cows	FBN_cows	1043	Carcass	YD	9
UAL (CA)	Angus, Charolais, Beef composite	Steers	UAL	7552	Growth and carcass	YD	5

*YD* yield deviation, *DYD* daughter yield deviation, *DRP* de-regressed proof

Overall, 28 different traits, measured in one to seven populations, were included in the meta-analyses (Table 2):

**Table 2:** Features of traits included in meta-analyses (MA)

Group of traits	Trait	Abbreviation	Number of populations^a^	MA^b^
Growth	Birth weight	BW	3	G1
Growth	Weight at 15 months	W15	1	G2
Growth	Weight at 18 months	W18	1	G2
Growth	Average daily gain	ADG	4	G2
Growth	Weight at slaughter	WS	4	G3
Live morphology	Muscularity score at 30 months	MS30	1	M1, M2
Live morphology	Muscularity of the thighs	THIGHS	2	M1
Live morphology	Muscularity of the withers	WITHER	2	M2
Live morphology	Length of the leg	LL	6	M3
Live morphology	Maximum width of the thigh	WT	5	M4
Live morphology	Skeletal score at 30 months	SS30	1	M5
Live morphology	Fat cover score	FS	1	C5
Carcass	Muscular development	MD	4	M1, M2
Carcass	Skeletal development	SD	3	M5
Carcass	Carcass weight	CW	7	C1
Carcass	Age at slaughter	AS	4	C2
Carcass	Carcass yield	CY	5	C3
Carcass	Carcass grade	CG	3	C4
Carcass	Lean meat yield	LMY	1	C4
Carcass	Meatiness	MT	1	C4
Carcass	Carcass conformation	CC	5	C4
Carcass	Fat coverage	FC	1	C5
Carcass	Ultrasound fat content	FCU	3	C5
Carcass	Carcass fat score	CFS	2	C5
Carcass	Average backfat thickness	ABT	1	C5
Carcass	Area of longissimus thoracis	ALT	5	C6
Carcass	Internal fat weight	IFW	5	C7
Carcass	Rib-eye area	REA	3	C8

^a^Number of populations in which the trait was measured

^b^Designations of the different meta-analyses (MA) focused on growth (G), morphology (M), and carcass traits (C) (see the description in Table 3)

- Five traits related to growth: weight at birth (BW), at month 15 (W15), at month 18 (W18), and at slaughter (WS), and average daily gain (ADG);
- Seven morphology phenotypes measured on the live animal: muscularity score at month 30 (MS30), skeletal score at month 30 (SS30), muscularity of the thighs (THIGHS), muscularity of the withers (WITHER), maximum width of the thigh (WT), leg length (LL), and fat score (FS);
- Sixteen carcass composition and conformation phenotypes: muscular development (MD), skeletal development (SD), carcass weight (CW), age at slaughter (AS), carcass grade (CG), carcass yield (CY), lean meat yield (LMY), meatiness (MT), carcass conformation (CC), fat coverage (FC), ultrasound fat content (FCU), carcass fat score (CFS), average backfat thickness (ABT), area of *longissimus thoracis* (ALT), internal fat weight (IFW), and rib eye area (REA).

Depending on the population, the traits were expressed as (1) yield deviations (YD), i.e., mean performance adjusted for environmental effects, (2) daughter yield deviations (DYD), only for bulls, i.e., mean performance of the daughters adjusted for environmental effects and the breeding value of their dam [10], or (3) de-regressed proof (DRP), i.e., theoretical phenotypes derived from estimated breeding values and their accuracies [11] (Table 1).

### Within-population sequence-based imputation and GWAS

Different partners conducted analyses in each population using similar methods for both imputation and GWAS. Physical coordinates of the variants were according to the ARS-UCD1.2 bovine genome assembly [12] and array-derived genotypes were imputed to the sequence level using a stepwise approach: (1) Illumina® 777k (high-density, HD) genotypes were imputed from 50k genotypes using version 5 of Beagle [13] or version 3 of FImpute [14], with 133 to 4059 purebred or multi-breed animals depending on the partner with HD genotypes as a reference (Table 3), and (2) sequence variants were imputed using 372 to 3093 animals from the RUN7 or RUN8 reference panel of the 1000 Bull Genomes consortium [5] with version 4 of Minimac [15] or version 5 of Beagle [13]. After filtering, 19.6 to 47.8 million biallelic variants were tested for association with different traits in each population in separate analyses using the GCTA software [16], accounting for a polygenic effect with a genomic relationship matrix estimated from 50k or high-density (HD) single nucleotide polymorphisms (SNPs). The following linear mixed model was applied:

**Table 3:** Software and reference population used for whole-genome sequence imputation

Population	Imputation software		Reference population		Number of animals	Number of biallelic variants
	Step 1 50k-HD	Step 2 HD-WGS	Step 1 50k-HD	Step 2 HD-WGS		
INRAE_CHA_cows	Fimpute3	Minimac4	672 CHA	1479 incl. 82 CHA	7999	25,050,323
INRAE_CHA_cows	Fimpute3	Minimac4	672 CHA	1479 incl. 82 CHA	8501	25,050,323
INRAE_MON_bulls	Fimpute3	Minimac4	530 MON	1479 incl. 63 MON	3226	25,050,323
INRAE_NOR_bulls	Fimpute3	Minimac4	551 NOR	1479 incl. 44 NOR	2749	25,050,323
INRAE_CHA_steers	Fimpute3	Minimac4	672 CHA	1479 incl. 82 CHA	4354	25,050,323
INRAE_MON_steers	Fimpute3	Minimac4	530 MON	1479 incl. 63 MON	4163	25,050,323
INRAE_NOR_steers	Fimpute3	Minimac4	551 NOR	1479 incl. 44 NOR	2730	25,050,323
INRAE-ULIM_CHA	Fimpute3	Minimac4	672 CHA	1479 incl. 82 CHA	1059	25,050,323
INRAE-ULIM_LIM	Fimpute3	Minimac4	462 LIM	1479 incl. 64 LIM	1209	25,050,323
INRAE-ULIM_BLA	Fimpute3	Minimac4	327 BLA	1479 incl. 41 BLA	939	25,050,323
ETH_bulls	Beagle5	Beagle5	1166 BSW and OBV	372 BSW and OBV	10,419	27,214,878
ETH_cows	Beagle5	Beagle5	1166 BSW and OBV	372 BSW and OBV	20,185	27,214,878
FBN_bulls	Beagle5	Beagle5	133 HOL and CHA F0 and F1 parents	1568 incl. 844 HOL and 144 CHA	1043	19,590,361
FBN_cows	Beagle5	Beagle5	133 HOL and CHA F0 and F1 parents	1568 incl. 844 HOL and 144 CHA	1043	19,590,361
UAL	Beagle5	Beagle5	4059 multi-breed	3093 *Bos taurus*	7552	47,833,012

*CHA* Charolais, *MON* Montbéliarde, *NOR* Normande, *LIM* Limousine, *BLA* Blonde d’Aquitaine, *BSW* Brown Swiss, *OBV* Original Braunvieh

1 $$\mathbf{y}=\mathbf{1}{\upmu }+\mathbf{x}\text{b}+\mathbf{g}+\mathbf{e},$$

where $\mathbf{y}$ is the vector of phenotypes (YD, DYD, or DRP); $\mathbf{1}$ is a vector of 1s; ${\upmu }$ is the overall mean; $\text{b}$ is the additive fixed effect of the variant tested; $\mathbf{x}$ is the vector of imputed allele dosages for the tested variant; $\mathbf{g} \sim N(\mathbf{0},\mathbf{G}{{\upsigma }}_{\text{g}}^{2})$ is the vector of random polygenic effects, with $\mathbf{G}$ the genomic relationship matrix based on 50k or HD autosomal SNPs, and ${{\upsigma }}_{\text{g}}^{2}$ the polygenic variance; and $\mathbf{e} \sim N(\mathbf{0},\mathbf{D}{{\upsigma }}_{e}^{2})$ is the vector of random residual effects, with ${{\upsigma }}_{e}^{2}$ the residual variance. $\mathbf{D}$ is the identity matrix for YD analyses and a diagonal matrix with inverse weights for DRP and DYD to account for heterogeneous accuracy.

### Quality control of summary GWAS

Before conducting meta-analyses, we performed quality control on the GWAS summary data. To ensure the consistency of trait measurements across different populations, we standardized the variant effects that were estimated by GCTA using the genetic standard deviation specific to each trait and population, as provided by each partner. We only retained bi-allelic variants [(SNPs or insertions-deletions (InDels)] that were imputed by at least two partners with concordant reference (REF)/alternative (ALT) alleles, resulting in 29.6 million variants. For each trait x population combination, our variant selection process involved multiple steps. First, we retained variants with a minor allele frequency (MAF) ≥ 0.005, except for populations from FBN with smaller cohorts, where we set the threshold at 0.02. This MAF threshold corresponded to approximately 10 to 200 minor allele counts, depending on the specific population. Furthermore, we filtered out variants with the lowest imputation accuracies. After comparing the imputation accuracy estimates from Beagle and Minimac—which overestimates and underestimates true R^2^, respectively (data not shown)—we decided to retain only the variants with an imputation R² value of ≥ 0.20. These filtering steps collectively resulted in the removal of approximately 25 to 50% of the initially considered variants, depending on the population. To further refine our dataset and avoid potential outliers, we excluded markers with absolute effects that exceeded five standard deviations of the trait distribution which were mostly observed for very rare variants. After filtering based on MAF and R², only a small fraction, ranging from 0.001 to 0.29%, were identified as outliers, depending on the population. For each retained variant, we checked that the allele used to compute doses in the different within-population GWAS was the same. In case of discordance, we changed the direction of the effect.

### Meta-analyses

From the 28 growth, morphology, and carcass traits with GWAS results from one to seven populations (Table 2), we formed 16 different groups for the meta-analyses: three for growth traits (G1–G3), five for morphology traits (M1–M5), and eight for traits measured on carcasses (C1–C8). When traits were relatively similar (e.g., weight at 15 months and weight at 18 months), they were grouped in the same meta-analysis. In each meta-analysis, no more than one trait was kept per population. Thus, each of the 16 meta-analyses combined results from one to five traits, three to ten populations, and 2636 to 25,367 animals (Table 4). After data filtering, between 17.9 and 24.9 million variants were analyzed in each MA.

**Table 4:** Traits and populations included in meta-analyses (MA)

MA	Group of traits	Trait^a^ (*populations*^b^)	Number of populations	Number of animals
G1	Growth	BW (*INRAE-ULIM_CHA, INRAE-ULIM_LIM, FBN_calves*)	3	2720
G2	Growth	W15 (*FBN_bulls*); W18 (*INRAE_CHA_cows*); ADG (*UAL, INRAE-ULIM_CHA, INRAE-ULIM_LIM, INRAE-ULIM_BLA*)	6	18,774
G3	Growth	WS (*INRAE-ULIM_LIM, INRAE-ULIM_BLA, FBN_bulls, FBN_cows*)	4	2636
M1	Morphology	MS30 (*INRAE_CHA_cows*); MD (*UAL, INRAE-ULIM_CHA, INRAE-ULIM_LIM, INRAE-ULIM_BLA*); THIGHS (*INRAE_MON_bulls, INRAE_NOR_bulls*)	7	17,418
M2	Morphology	MS30 (*INRAE_CHA_cows*); MD (*UAL, INRAE-ULIM_CHA, INRAE-ULIM_LIM, INRAE-ULIM_BLA*); WITHER (*INRAE_MON_bulls, INRAE_NOR_bulls*)	7	17,418
M3	Morphology	LL (*UAL, INRAE-ULIM_CHA, INRAE-ULIM_LIM, INRAE-ULIM_BLA, FBN_bulls, FBN_cows*)	6	3695
M4	Morphology	WT (*INRAE-ULIM_CHA, INRAE-ULIM_LIM, INRAE-ULIM_BLA, FBN_bulls, FBN_cows*)	5	3695
M5	Morphology	SD (*INRAE-ULIM_CHA, INRAE-ULIM_LIM, INRAE-ULIM_BLA*); SS30 (*INRAE_CHA_cows*)	4	12,140
C1	Carcass	CW (*INRAE_CHA_steers, INRAE_MON_steers, INRAE_NOR_steers, FBN_bulls, FBN_cows, ETH_bulls, UAL*)	7	19,989
C2	Carcass	AS (*INRAE_CHA_steers, INRAE_MON_steers, INRAE_NOR_steers, INRAE-ULIM_CHA*)	4	12,208
C3	Carcass	CY (*INRAE-ULIM_CHA, INRAE-ULIM_LIM, INRAE-ULIM_BLA, FBN_bulls, FBN_cows*)	5	3694
C4	Carcass	CG (*INRAE_CHA_steers, INRAE_MON_steers, INRAE_NOR_steers*); LMY (*UAL*); MT (*ETH_bulls*); CC (*INRAE-ULIM_CHA, INRAE-ULIM_LIM, INRAE-ULIM_BLA, FBN_bulls, FBN_cows*)	10	25,367
C5	Carcass	FS (*INRAE_NOR_bulls*); FCU (*INRAE-ULIM_CHA, INRAE-ULIM_LIM, INRAE-ULIM_BLA*); CFS (*FBN_bulls, FBN_cows*); ABT (*UAL*); FC (*ETH_bulls*)	8	14,622
C6	Carcass	ALT (*INRAE-ULIM_CHA, INRAE-ULIM_LIM, INRAE-ULIM_BLA, FBN_bulls, FBN_cows*)	5	3692
C7	Carcass	IFW (*INRAE-ULIM_CHA, INRAE-ULIM_LIM, INRAE-ULIM_BLA, FBN_bulls, FBN_cows*)	5	3686
C8	Carcass	REA (*UAL, FBN_bulls, FBN_cows*)	3	4453

*MA* meta-analysis

^a^See the description of the traits in Table 2

^b^See the description of the populations in Table 1

For the MA, we used both z-score and fixed-effects methods implemented in the METAL software [17]. Each of these methods combines different parameters from individual analyses: sample size, p-values (*p*_i_), and direction of effects for the z-score method and effect estimates and standard errors for the fixed-effects method. For each variant, the z-score method converts the p-value into a z-score $Z={{\Sigma }}_{i}{z}_{i}{w}_{i}/\left({{\Sigma }}_{i}{w}_{i}^{2}\right)$, where ${w}_{i}$ is the square root of the sample size for study $i$ and ${z}_{i}={{\Phi }}^{-1}(1-{p}_{i}/2)\times (\text{effect}\; \text{direction}\; \text{for}\; \text{study}\; i)$, with ${\Phi }$ the cumulative distribution function of a normal distribution and ${p}_{i}$ the $p$-value of the $i\text{th}$ study. The fixed-effects method assumes that the true effect of each allele is the same across the different studies and combines effects by weighting them by the inverse of their standard errors. Therefore, both MA methods weight the different studies by their sample size [8].

### Definition of QTL

For all GWAS or MA results, we applied a uniform threshold (− log_10_(*p*_*i*_) = 8.7) corresponding to a 5% genome-wide threshold of significance after Bonferroni correction for ~ 25 million variants. Bounds of the confidence intervals (CI) of the QTL were defined by the locations of the most distant variants present in the upper third of the peak at ± 2 Mb around the lead variant, i.e., the variant with the most significant effect. This method was found to be more reliable than the constant LOD drop-off method [18], especially for high values of test statistics [19, 20].

### Post-GWAS analyses

Variants located in the CI of the QTL were functionally annotated with the Ensembl variant effect predictor (VEP) pipeline v81 [21].

QTL annotation and enrichment analyses were performed using the R package GALLO [22], based on lists of variants in the CI of the QTL detected in within-population GWAS, and fixed-effects and z-score MA. To determine if certain types of QTL were under- or over-represented within the genome, we compared the results of the present study with the number of QTL types referenced in CattleQTLdb (https://www.animalgenome.org/cattle) [23]. Among the QTL types, the top enriched ones with a false discovery rate lower than 0.05 were retained.

Finally, we assessed whether variants in the CI of the QTL co-localized with regulatory variants with a significant effect (adjusted p-value for multiple testing < 0.05) on the expression (cis-eQTL) and alternative splicing (cis-sQTL) of neighboring genes (cis) in 23 bovine tissues (tissues referenced as *Muscle*, *Muscle_taurus, Muscle_indicus*, and *Muscle_cross* were gathered in a single *Muscle* group) available in the CattleGTEx database (https://cgtex.roslin.ed.ac.uk) [23]. For the enrichment analyses and the visualization of results, we used the eQTpLot R package, which was first developed in humans for the visualization of colocalization between eQTL and GWAS results [24] and adapted to bovine data for the present study. Both *PanTissue* (considering eQTL results for all 23 tissues) and *MultiTissue* (focused on the nine following targeted tissues: *Adipose, Muscle_indicus, Intramuscular_fat, Muscle, Muscle_taurus, Liver, Muscle_cross, Pituitary, Hypothalamus*) analyses were performed. Variants with significant effects on both (i) beef traits in MA of the present study (QTL, with an adjusted p-value for Bonferroni correction < 0.05, i.e., a nominal p-value < 2.10^−9^) and (ii) gene expression in CattleGTEx (eQTL, with an adjusted p-value for multiple testing by permutation < 0.05) were considered for the enrichment analyses and the visualization of results. For eQTL, we applied the default method that retained the most significant eQTL, i.e., the one with the lowest p-value among all the selected tissues.

## Results

### Comparison of the QTL detected in within-population GWAS and meta-analyses

QTL were detected in 15 of the 16 MA with both the fixed-effects and z-score methods, and in one to five of the constituent within-population GWAS that made up the MA (Table 5). With the exception of the G1 and C3 MA, both fixed-effects and z-score MA identified at least as many QTL as any of the within-population GWAS that were included in the MA. In total, 101 and 93 QTL were identified with the fixed-effects and z-score methods, i.e., an average of 6.7 and 6.2 QTL per MA, respectively. Moreover, the CI of the QTL detected with these methods were shorter (fixed-effects mean: 77 variants in 605 kb; z-score mean: 106 variants in 1582 kb) than those detected in within-population GWAS (218 variants in 1810 bp on average).

**Table 5:** Features of QTL identified in within-population GWAS and meta-analyses (MA)

MA^a^	Within-population GWAS				Fixed-effects MA			Z-score MA		
	Number of QTL total	Number of QTL per GWAS^b^	Number of variants (CI)	Size in kb (CI)	Number of QTL	Number of variants (CI)	Size in kb (CI)	Number of QTL	Number of variants (CI)	Size in kb (CI)
G1 (3)	8	8	135 (12–381)	1650 (415–2448)	6	124 (44–223)	1736 (569–3594)	4	32 (3–81)	1691 (389–3245)
G2 (6)	8	4,4	216 (22–707)	1823 (629–2903)	8	50 (14–100)	1310 (104–2828)	8	56 (5–128)	896 (34–2828)
G3 (4)	6	4,1,1	215 (31–564)	2441 (1183–3922)	8	114 (5–419)	1696 (129–3426)	7	75 (5–366)	1490 (760–2143)
M1 (7)	15	1,6,2,6	153 (3–566)	1438 (52–2863)	8	81 (2–429)	1070 (109–2863)	8	79 (3–384)	860 (49–2863)
M2 (7)	11	1,6,2,2	170 (3–566)	1526 (109–3101)	8	60 (2–411)	882 (49–2863)	7	62 (2–382)	721 (49–2863)
M3 (6)	12	2,8,1,1	271 (17–793)	1937 (495–3823)	9	102 (4–421)	1519 (274–3038)	8	82 (12–376)	1883 (693–3215)
M4 (5)	8	1,3,3,1	333 (32–800)	2357 (1417–3578)	6	161 (5–409)	1701 (559–2386)	7	108 (3–383)	1328 (340–2300)
M5 (4)	12	1,10,1	182 (18–507)	1871 (416–3511)	11	125 (2–347)	1611 (146–2997)	13	86 (4–321)	1724 (158–3025)
C1 (7)	3	3	66 (34–126)	469 (174–823)	4	35 (7–87)	928 (78–1443)	4	16 (2–52)	338 (37–627)
C3 (5)	15	7,2,4,2	290 (4–902)	1840 (59–3821)	6	237 (71–850)	2392 (721–3614)	5	179 (11–637)	1782 (721–3419)
C4 (10)	11	3,1,3,3,1	207 (3–570)	1668 (680–2520)	8	33 (3–161)	1272 (24–2520)	8	15 (3–34)	1186 (69–2520)
C5 (8)	5	3,1,1	128 (1–403)	1547 (0–2664)	6	42 (1–90)	1025 (0–2623)	4	22 (1–45)	902 (0–2401)
C6 (5)	8	3,2,3	176 (9–766)	1869 (1–3143)	5	266 (49–474)	1975 (935–2577)	4	177 (8–366)	1780 (909–2300)
C7 (5)	1	1	533	2205	1	95	2857	1	80	2575
C8 (3)	6	4,1,1	195 (17–445)	2515 (989–3892)	7	66 (3–232)	1750 (98–3982)	5	80 (2–234)	2174 (586–3748)
Mean	8.6		218	1810	6.7	106	1582	6.2	77	605

*CI* confidence interval

^a^Meta-analysis (MA) and number of constituent within-population GWAS in brackets

^b^Number of QTL detected in each within-population GWAS with significant results

Only two QTL detected in a within-population GWAS for a particular trait were not found in any of the MA. These QTL, detected in the INRAE-CHA-COWS population for W18 and in the INRAE-ULIM-STEERS population for CY, were located on *Bos taurus* (BTA) autosomes 25 (~ 1 Mb) and 10 (~ 59 Mb), respectively. In contrast, nine QTL were identified in at least one MA that had not been found in any of the corresponding within-population GWAS, which demonstrate that MA can increase the power to detect association signals. These nine QTL were detected in the following MA: G2 (BTA5 ~ 106 Mb), M1 (BTA7 ~ 90.9 Mb), M2 (BTA7 ~ 90.9 Mb and BTA14 ~ 23.3 Mb), C1 (BTA2 ~ 6.3 Mb and BTA10 ~ 59 Mb), C4 (BTA10 ~ 59 Mb), C5 (BTA17 ~ 60.4 Mb), and C6 (BTA6 ~ 37.3 Mb). In addition, for the QTL that were identified in both a within-population GWAS and in an MA, the QTL detected in the MA had a more significant effect than the one detected in the corresponding within-population GWAS (see Additional file 1: Table S1).

However, functional annotation revealed no major differences between the variants located in the CI of the QTL identified in within-population GWAS, fixed-effects MA, and z-score MA (Table 6). In all cases, intergenic variants were the most frequent (55, 63, and 61%, respectively), followed by intronic variants (30, 24, and 25%, respectively). Depending on the analysis, between 5.5 and 6.6% of variants were located in the upstream or downstream regions of genes. However, regardless of the analysis considered, less than 1% of the variants were located in the 3′UTR or 5′UTR regions or in protein coding regions (e.g., synonymous, missense, stop-gain variants).

**Table 6:** Distribution (%) of functional annotations of variants within the CI of QTL regions identified in within-population GWAS, and in fixed-effects and z-score meta-analyses (MA)

Functional annotation	Within-population GWAS	Fixed-effects MA	z-score MA	Total
Intergenic_region	55.0	63.0	61.0	57.8
Intron_variant	30.3	24.2	25.2	28.1
Upstream_gene_variant	6.6	5.5	6.0	6.3
Downstream_gene_variant	6.1	5.6	5.7	5.9
Missense_variant	0.53	0.68	0.69	0.59
3_prime_UTR_variant	0.49	0.40	0.59	0.49
Synonymous_variant	0.44	0.23	0.24	0.36
5_prime_UTR_variant	0.22	0.27	0.30	0.25
Splice_region_variant and intron_variant	0.079	0.040	0.015	0.060
Stop_gained	0.053	0.101	0.136	0.077
Frameshift_variant	0.045	0.050	0.075	0.051
Non_coding_transcript_exon_variant	0.023	0.020	0.030	0.023
5_prime_UTR_premature_start_codon_gain_variant	0.011	0.010	0.000	0.009
Splice_region_variant and synonymous_variant	0.011	0.000	0.000	0.007
Stop_lost and splice_region_variant	0.004	0.000	0.000	0.002
Gene_fusion	0.000	0.000	0.015	0.002

*CI* confidence interval, *MA* meta-analysis

### Features of the QTL identified in meta-analyses

**Fig. 1:**
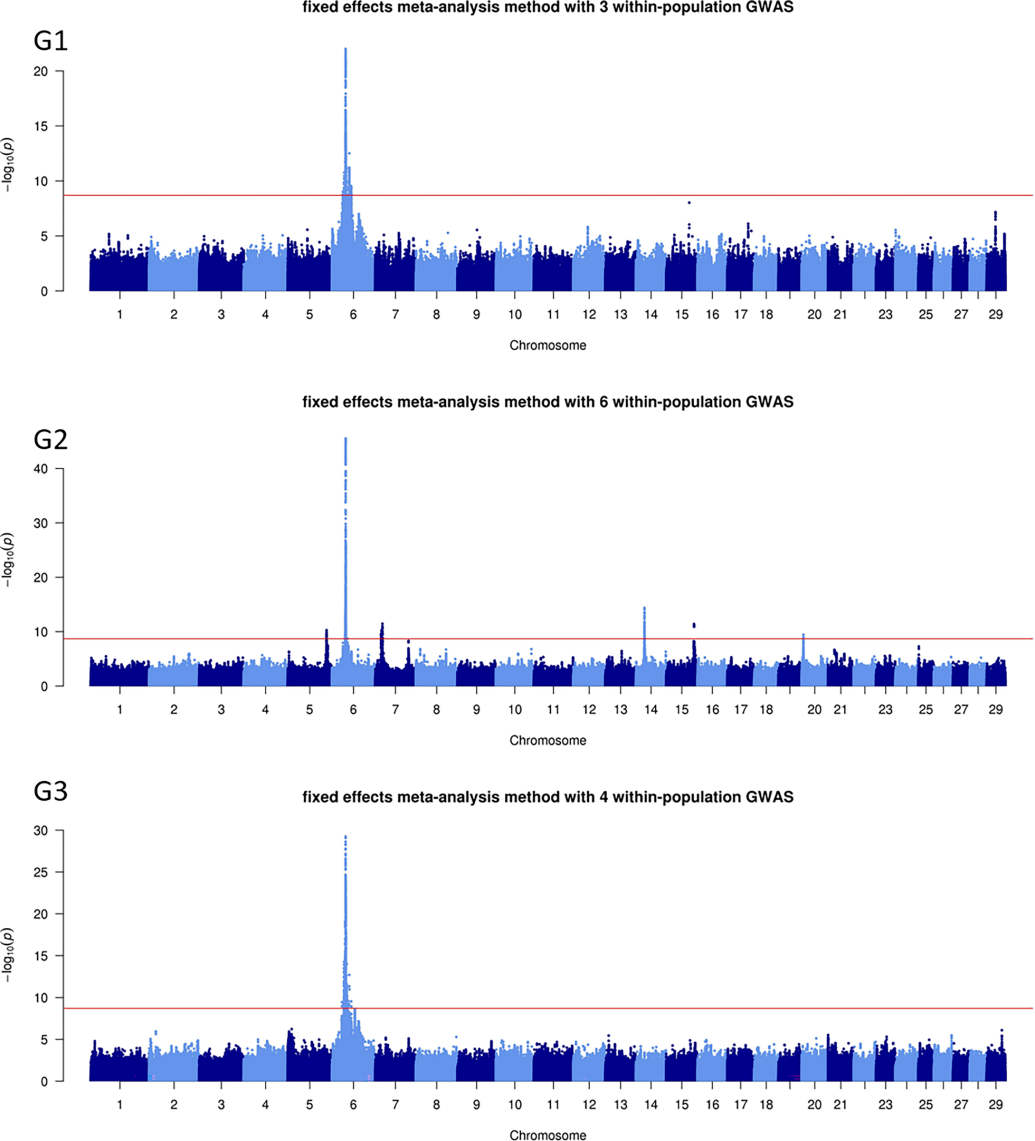
− log10(P) values plotted against the position of variants on *Bos taurus* (BTA) autosomes for the meta-analyses of growth traits with the fixed-effects method. See Tables 2 and 4 for traits and populations included in the G1, G2, and G3 growth meta-analyses

**Fig. 2:**
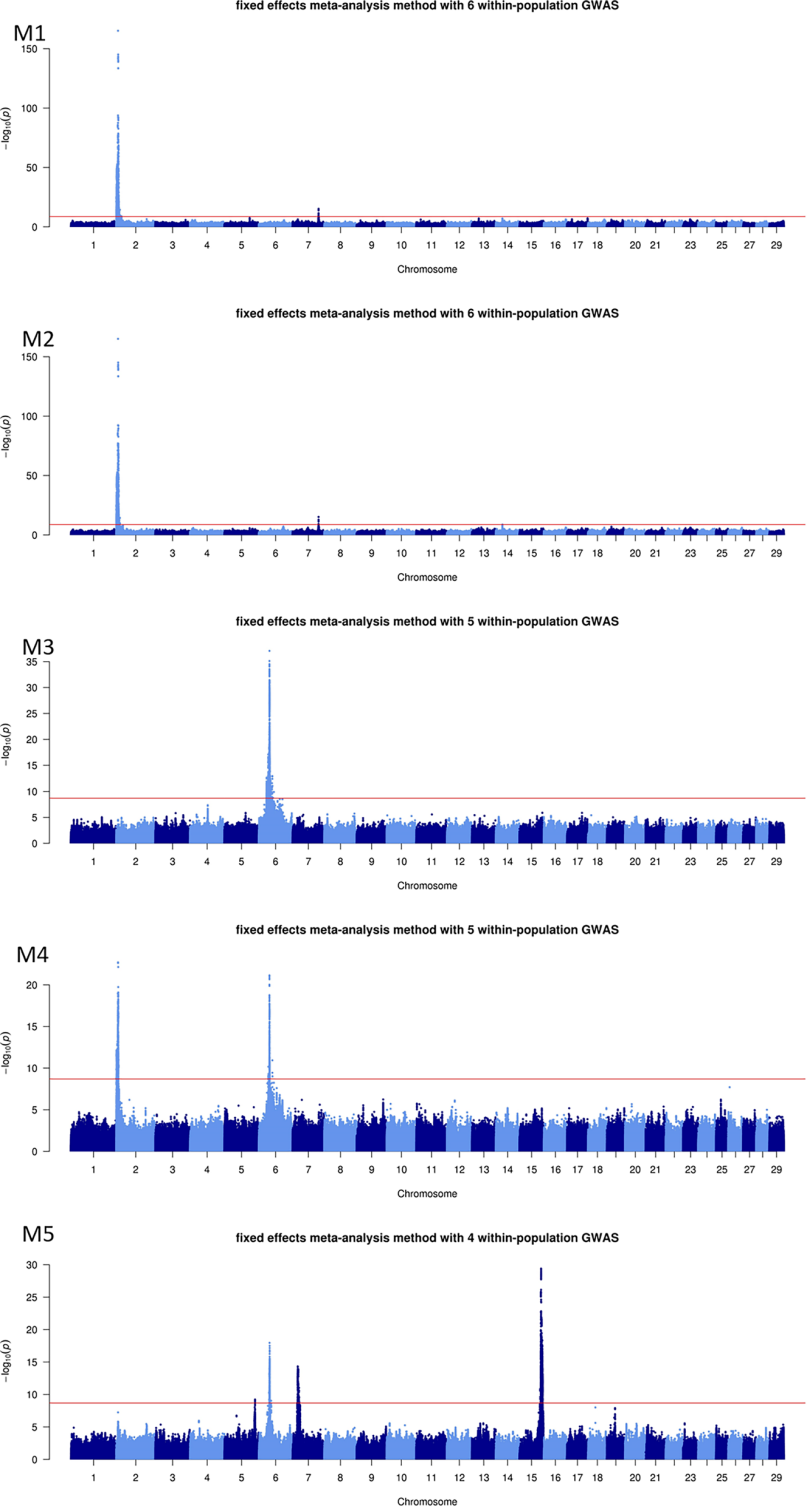
− log10(P) values plotted against the position of variants on *Bos taurus* (BTA) autosomes for the meta-analyses of morphology traits with the fixed-effects method. See Tables 2 and 4 for traits and populations included in the M1, M2, M3, M4, and M5 meta-analyses

**Fig. 3:**
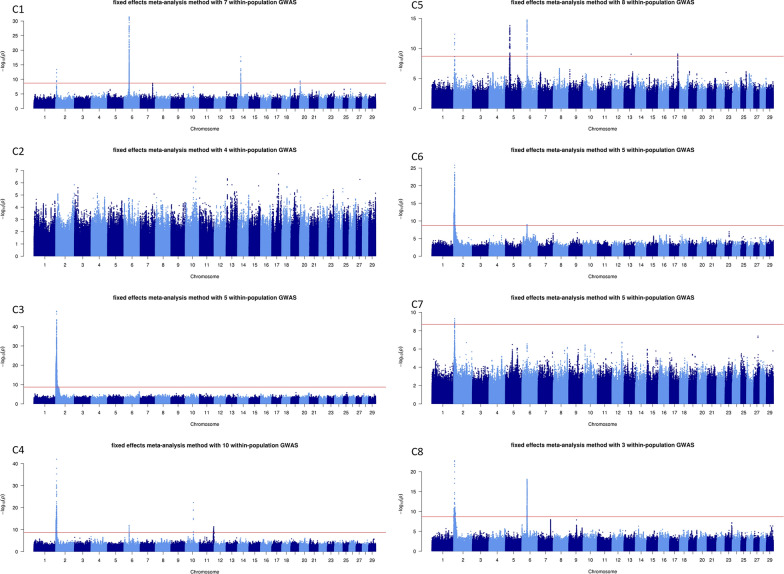
− log10(P) values plotted against the position of variants on *Bos taurus* (BTA) autosomes for the meta-analyses of carcass traits with the fixed-effects method. See Tables 2 and 4 for traits and populations included in the C1, C2, C3, C4, C5, C6, C7, and C8 meta-analyses

The number of QTL varied greatly depending on the MA and method used, from one QTL identified with both methods for C7 (carcass IFW) to 11 and 13 QTL with the fixed-effects and z-score methods, respectively, for M5 (skeletal development). Overall, the results obtained with the two MA methods were similar, although the fixed-effects MA tended to detect more QTL than the z-score MA. Consequently, we present here only the Manhattan plots for the fixed-effects MA for growth traits (G1, G2, and G3; Fig. 1), morphology traits (M1, M2, M3, M4, and M5; Fig. 2), and carcass traits (C1, C2, C3, C4, C5, C6, C7, and C8; Fig. 3). For more details, see Manhattan plots for each within-population GWAS and both fixed-effects and z-score MA methods (see Additional file 2: Fig. S1) and features of the QTL detected in the fixed-effects and z-score MA (see Additional file 1: Table S1).

### Growth traits

In the three MA related to growth traits, the QTL with the most significant effect was the same, located on BTA6 at around 37 Mb (both fixed-effects and z-score). In each of these three MA, the lead variant differed between fixed-effects and z-score MA: rs109732906 and rs383507085 in G1, rs381322509 and rs109732906 in G2, and rs109732906 and rs110995268 in G3, respectively. In all cases, the fixed-effects MA identified the lead variant with the most significant effect. Notably, the rs109732906 variant, located in an intron of the *LCORL* gene, was a lead variant in each MA related to growth traits. In addition to the QTL detected on BTA6, the G2 MA also found QTL on BTA5, 7, 14, 15, and 20, but the effects of these QTL were much less significant than the one detected on BTA6. The lead variants on BTA5, 7, and 14 were located in the *CCDN2*, *PIAS4*, and *PLAG1* genes, respectively, in both MA methods.

### Morphology traits

In the five MA conducted on morphology traits, we identified 42 and 43 QTL using the fixed-effects and z-score methods, respectively. The QTL with the most significant effects were located on BTA2, 6, and 15. The lead variant identified on BTA2 in M1 and M2 (both methods) and in M4 (fixed-effects only) was a stop-gain variant in the *MSTN* gene (rs110344317). Instead, in M4, the z-score method identified *ANKAR* as the best candidate gene on BTA2. On BTA6, a QTL with a very significant effect was identified in the vicinity of the *LCORL* gene. However, the lead variants, although still intronic, differed depending on the MA and method considered: rs109114124 in M3 and M5 with both methods, and rs110995268 and rs109256415 in M4 with the fixed-effects and z-score methods, respectively. Fixed-effects and z-score methods pointed to two different, but close, lead variants on BTA15 in the M5 MA: rs801276339 in the downstream region of the *SLC35C1* gene and rs451134493 in an intron of the *CRY2* gene, respectively. Other candidate genes targeted in the different MA included genes on BTA2 (*CYFIP1*, *SESTD1*, *HS6ST1*, *HERC2*, and *CCDC141*), BTA6 (*SEL1L3*, *CCSER1*, *GRID2*, *KCNIP4*, and *SLIT2*), BTA7 (*MLLT1*, *TJP3*, *KHSRP*, and *NMRK2*), and BTA14 (*PLAG1*).

### Carcass traits

The results of three to ten within-population GWAS on carcass traits were combined into eight different MA that led to the identification of 37 and 41 QTL with the fixed-effects and z-score methods, respectively. These QTL were distributed on BTA2, 5, 6, 10, 11, 13, 14, 17, and 20. The QTL with the most significant effects were located on BTA2 and 6 in the same regions highlighted for their effects on growth and morphology traits. However, on BTA2, the lead variants were located in different genes from those of the variants detected in the earlier analyses (e.g., rs110344317 in *MSTN*, rs208026566 in *WDR75*, rs109522136 in *HERC2*, rs798066180 in *COL3A1*, and a variant at 6,601,383 bp in *ANKAR*). Instead, on BTA6, the lead variants were located in or close to the same gene, *LCORL*, highlighted for its effects on growth and morphology. Among the lead variants found in the *LCORL* gene, three were intronic variants (rs109188585, rs109696064, and rs381823183), one was a frameshift variant (rs384548488), and another was a missense variant (rs109696064). Other regions with significant effects were identified on BTA5 (*SPATS2* and *FAIM2*), 11 (*MED27* and *CACNA1B*), 13 (*MRPS26*), 14 (*PLAG1*), and 20 (*ERGIC1*). For three of these genes, the lead variants were located in either the 3′UTR region (rs41933328 in *ERGIC1* and rs470093266 in *FAIM2*) or the 5′UTR region (rs210030313 in *PLAG1*) and could therefore potentially modulate the expression of these genes.

### Comparison with QTL from the literature

**Fig. 4:**
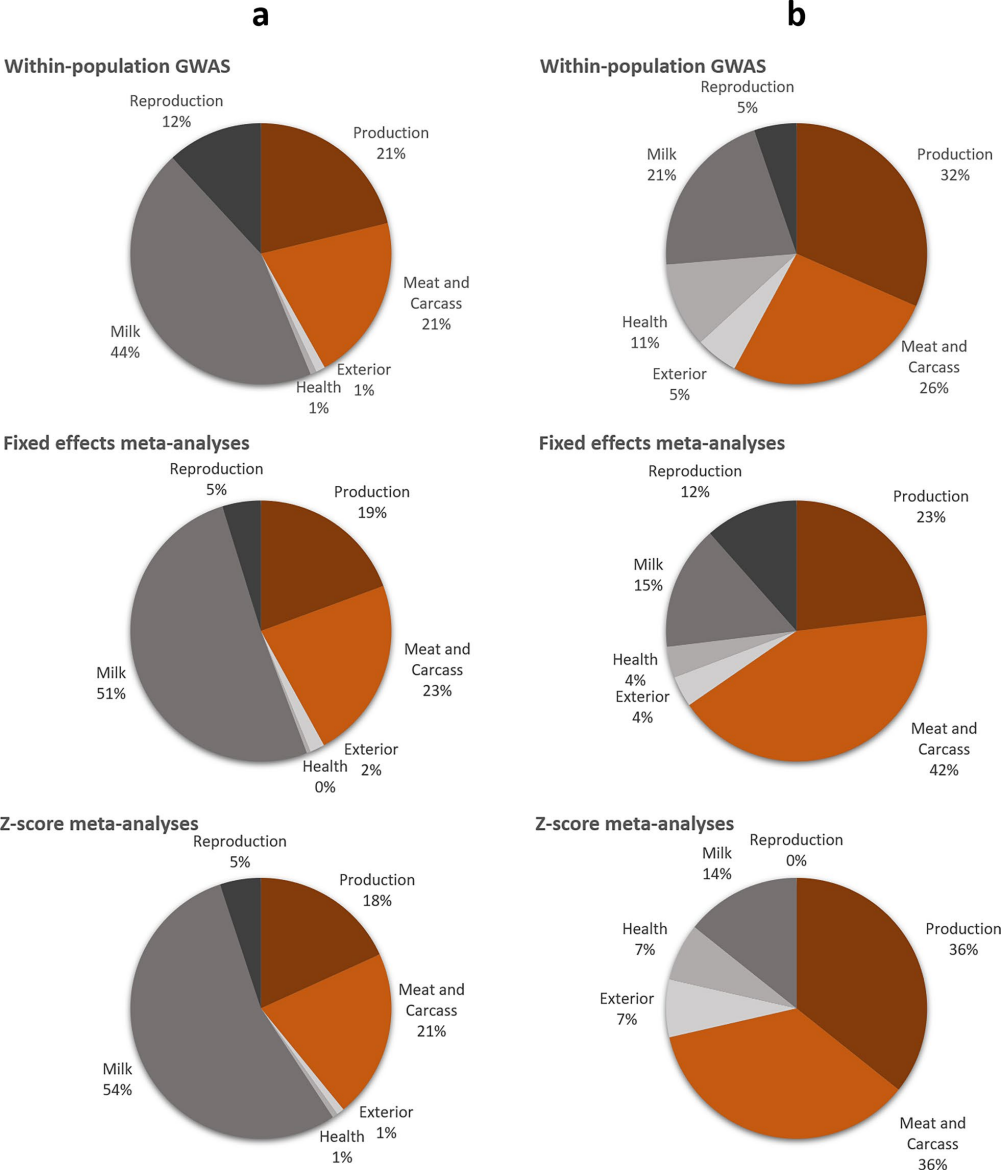
Results of QTL enrichment analyses. **a** AnimalQTLdb annotation of variants located within confidence intervals of the QTL. **b** QTL enrichment analyses (p-value adjusted FDR < 0.05).

We assembled lists of all the variants located within the CI of the QTL detected in the within-population GWAS (n = 13,060), the fixed-effects MA (n = 5611), and the z-score MA (n = 3797) and compared these with QTL from CattleQTLdb with a reported effect in cattle. The three sets of variants identified in our study represented, respectively, 72, 65, and 63 QTL regions that had previously been noted for their effects on milk (milk protein and fatty acid composition), production (weight and average daily gain), meat and carcass traits, reproduction, health, and exterior type traits (udder swelling score) (see Additional file 3: Table S2). These categories accounted for, respectively, 44%, 21%, 21%, 12%, 1%, and 1% of QTL in the within-population GWAS; 51%, 19%, 23%, 5%, 0%, and 2% of QTL in the fixed-effects MA; and 54%, 18%, 21%, 5%, 1%, and 1% of QTL in the z-score MA (Fig. 4a). However, in the cattle database, a disproportionate number of QTL were associated with milk-related traits; to address this bias, we performed a QTL enrichment analysis that compared the number of QTL identified within the candidate regions with the number of QTL in CattleQTLdb. These analyses revealed 19, 26, and 14 significantly enriched traits (adjusted p-value < 0.05) for within-population GWAS, fixed-effects MA, and z-score MA, respectively. For the three different analyses, these traits were found to be linked with milk (21%, 15%, and 14%), production (32%, 23%, and 36%), meat and carcass traits (26%, 42%, and 36%), reproduction (5%, 12%, and 0%), health (11%, 4%, and 7%), and exterior traits (5%, 4%, and 7%), respectively (Fig. 4b). Therefore, an enrichment of meat/carcass and production QTL was observed in the within-population GWAS (58%) and even more so in the MA (65% and 72% with the fixed-effects and z-score methods, respectively).

### Overlapping between QTL detected in MA and e/sQTL

An examination of co-localization between the QTL detected in the MA of the current study and the cis-e/sQTL available in CattleGTEx revealed 54 eQTL × tissue × MA × method combinations (Table 7) and 170 sQTL × tissue × MA × method combinations (Table 8). Variants located in the CI of QTL from ten MA were identified as eQTL (32 variants with fixed-effects and 22 with z-score), while those from 14 MA were identified as sQTL (91 variants with fixed-effects and 71 with z-score). Depending on the trait analyzed and the MA method used, the number of eQTL and sQTL ranged from 0 to 34. The largest amount of co-localization between the QTL of this study and e/sQTL was identified for the M5 MA, which focused on the skeletal development of animals (9 and 8 eQTL and 32 and 34 sQTL for the fixed-effects and z-score methods, respectively). From these e/sQTL, we identified 16 and 22 tissues or cell lines in which the expression or the alternative splicing of a gene was affected, respectively. The largest number of regulatory variants co-localizing with QTL was identified in muscle tissue and leukocytes (eQTL) and in adipose tissue (sQTL).

**Table 7:** Number of eQTL variants in the CI of QTL detected with the fixed-effects/z-score MA methods in different tissues and MA

Tissue	G1	G2	G3	M2	M3	M5	C3	C5	C6	C8	Total
Adipose	1/0					0/1				1/1	2/2
Blood									1/0		1/0
Embryo				1/0							1/0
Intramuscular_fat						1/1					1/1
Leukocyte	1/0		1/1		1/1	0/1				1/1	4/4
Liver			1/1		0/1	1/0					2/2
Lung						0/1					0/1
Lymph_node		0/1				3/0		1/0			4/1
Mammary						1/1					1/1
Mammary_L						1/1					1/1
Milk_cell	1/0										1/0
Monocytes		0/1	1/1		0/1				1/0		2/3
Muscle	1/0					1/2	1/1		2/0		5/3
Pituitary		0/1									0/1
Rumen	1/0				1/0						2/0
Spleen			1/1		1/1	1/0		1/0	1/0		5/2
Total	5/0	0/3	4/4	1/0	3/4	9/8	1/1	2/0	5/0	2/2	32/22

*CI* confidence interval, *MA* meta-analysis

**Table 8:** Number of sQTL variants in the CI of QTL detected with the fixed-effects/z-score MA methods in different tissues and MA

Tissue/MA	G1	G2	G3	M1	M2	M3	M4	M5	C1	C3	C5	C6	C7	C8	Total
Adipose	2/0		2/2			2/2		4/2				1/0			11/6
Blood		0/1		1/0				0/4	1/0			1/0			3/5
Embryo												2/0			2/0
Hypothalamus		0/1						2/1			1/0	1/0			4/2
Jejunum								2/0				2/0			4/0
Kidney		0/1						4/3				1/0			5/4
Liver					1/0			2/0		1/0	1/0	1/0			6/0
Lung	2/0	1/1			1/0			3/1		1/0		1/0			9/2
Lymph_node		1/1				0/1		2/5							3/7
Macrophage	1/0	1/3		1/1	1/0			2/0							6/4
Mammary	1/0	0/1	2/2			2/2		1/1				1/0			7/6
Mammary_L		0/2					0/1	1/3						1/1	2/7
Milk_cell		0/1						0/2				1/0			1/3
Monocytes					0/1			0/1							0/2
Muscle		0/1				0/4		2/3		1/0		1/0			4/8
Ovary										1/0					1/0
Oviduct		0/1	1/1			1/1	0/1			1/1		1/1	1/1		5/7
Pituitary	1/0	1/0				0/1		1/2							3/3
Rumen			1/1			1/1		2/2							4/4
Spleen		1/1	1/1			1/1		1/2				1/0			5/5
Testis		0/1	0/1					2/2				2/0			4/4
Uterus								1/0		1/0					2/0
Total	7/0	5/16	7/8	2/1	3/1	7/13	0/2	32/34	1/0	6/1	2/0	17/1	1/1	1/1	91/79

**Fig. 5:**
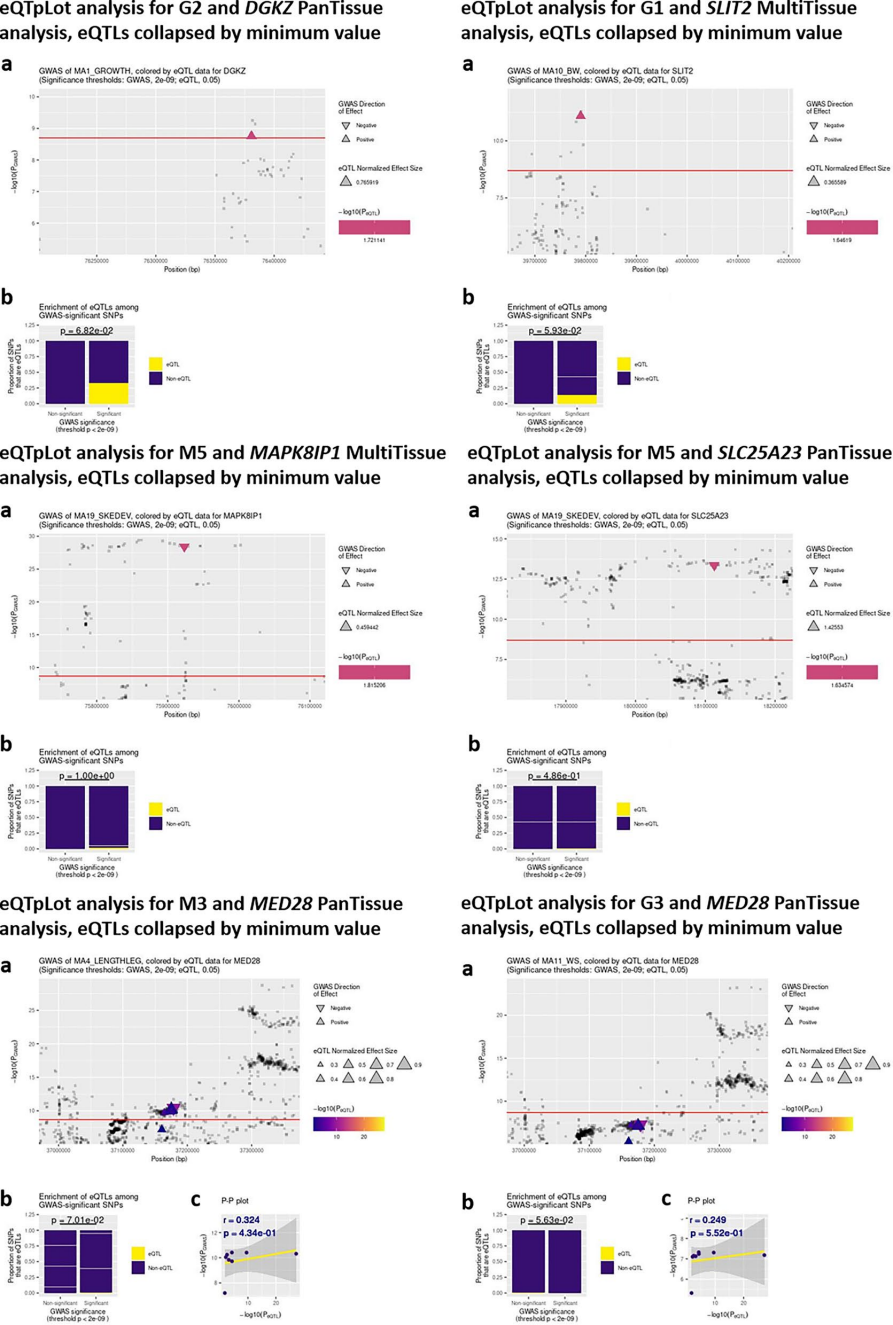
Co-localization between QTL found in meta-analyses and eQTL from CattleGTEx (eQTpLot results). **a** GWAS results colored by eQTL results. **b** Enrichment of eQTL among GWAS-significant variants. **c** − log(P_QTL_) plotted against − log(P_eQTL_) when relevant, i.e., when several variants presented significant results in both QTL and eQTL analyses

In two MA related to growth traits—G1 (BW) and G2 (W15, W18, and ADG)—we identified an eQTL among the variants with the most significant effects; these eQTL had effects on the expression of *SLIT2* (BTA6) and *DGKZ* (BTA15), respectively. In the M5 MA, the QTL/eQTL co-localization study highlighted two variants that affect the expression of *MAPK8IP1* (BTA15) and *SLC25A23* (BTA5), which were among those with the most significant effects in the four QTL regions (Fig. 5). In two other MA (G3 for WS and M3 for LL), we found several variants in a QTL region located on BTA6 that have been reported to regulate the expression of *MED28*. In both cases, we observed a tendency for eQTL to be overrepresented in the lists of significant variants from the MA (p-value = 0.06 for G3 and p-value = 0.07 for M3), as well as a slightly positive correlation between the − log10(p-value) obtained in QTL and eQTL studies (r = 0.25 for G3 and r = 0.32 for M3). However, the variants that were highlighted in this region were not among the variants with the most significant effects (Fig. 5).

## Discussion

This study, conducted on imputed whole-genome sequences of 54,782 animals from 15 populations of various breeds, is the first meta-analysis of this scale dedicated to beef cattle production, while GWAS results from large cohorts have been reported before [25, 26]. It demonstrates the value of meta-analyses as a complement to within-population GWAS, in identifying (i) a larger number of QTL, (ii) QTL with more significant effects, (iii) a smaller number of target variants within the CI of the QTL, and (iv) a more significant enrichment of meat, carcass, and production QTL. In terms of power and mapping precision, the superiority of meta-analyses over GWAS may be due to both the larger number of animals used and the fact that, between breeds, LD extends over shorter distances. This study employed the two most commonly used meta-analysis methods for GWAS, i.e., z-score and fixed-effects approaches, and confirms that, although the different meta-analyses in this study combined substantially different traits, the fixed-effects method appears to be more powerful in detecting QTL [7]. The rare QTL (n = 2), identified in within-population GWAS but absent in MA, may correspond to specific QTL that are not shared between the populations analyzed in the present study. Alternatively, they might represent shared QTL with different causal mutations. In either scenario, the MA tends to weaken the signal rather than amplify it.

Growth, morphology, and carcass traits have been extensively studied in cattle. The QTL enrichment analyses performed in this study revealed a highly significant enrichment of QTL referenced in CattleQTLdb [22] for these traits. The database contains more than 15,000 QTL recorded for growth traits, of which almost half are located on BTA6, 7% on BTA14, 5.3% on BTA20, and between 0.7 and 3.7% on the other chromosomes. For morphology and carcass traits, which are more difficult to measure, a significantly smaller number of QTL (n = 878) are described in CattleQTLdb with effects on anatomy, fatness, or carcass quality. In contrast to growth traits, QTL linked with morphology and carcass traits are more evenly distributed among the different autosomes (from 0.8 to 8.4%), with BTA 2 (8.4%), 5 (8%), 14 (7.9%), and 13 (6.9%) having the largest number. Overall, the results of the present meta-analyses are very consistent with the QTL described in the literature. Indeed, using the fixed-effects meta-analysis method, we found 38 QTL on BTA2 (only in morphology and carcass MA), 36 on BTA6 (in growth, morphology, and carcass MA), 7 on BTA7, 5 on BTA15, 3 on BTA11 and 14, 2 on BTA20, and 1 each on chromosomes 10, 13, and 17. Therefore, this study confirms previous research that the number of QTL found is much larger on chromosomes 2 (at ~ 6.3 Mb) and 6 (at ~ 37.6 Mb) than on other bovine autosomes [25–28].

A recent study reported that variants that influence gene expression or RNA splicing can account for a significant proportion of the heritability of complex traits in cattle [29]. To investigate this, we compared the results of our QTL meta-analyses with cis-e/sQTL results available in CattleGTEx [23]. Our analysis identified a limited number of variants that may affect beef traits by regulating gene expression or RNA splicing. To reduce the risk of false positives, we excluded trans-e/sQTL, which accounted for most of the heritability of the traits studied by Xiang et al. [29]; this may have contributed to the limited number of regulating variants identified in our analysis. Nonetheless, this approach enabled us to identify promising candidate genes, including *MED28*, *DGKZ*, *SLIT2*, *MAPKBIP1*, and *SLC25A23*, which were not explicitly highlighted in the GWAS meta-analyses.

On BTA2, the *MSTN* gene encodes myostatin, a component of transforming growth factor β (TGF-β). This gene has been known to impact muscle growth, for decades [30], and has been found to be associated with morphology and carcass traits in many studies conducted in different breeds of cattle [30–35]. Several mutations in the *MSTN* gene have been reported to affect the muscularity of animals. Of note, the Q204X mutation, which causes a premature stop codon in *MSTN* and was ranked 1st in the QTL peaks with the most significant effects in our study (− log_10_(p-value) = 165), has been reported as one of the polymorphisms responsible for muscular hypertrophy, also known as the double-muscled phenotype, in several cattle breeds [36]. The Q204X mutation was also the lead variant at a QTL identified in the within-population GWAS performed with purebred or crossbred Charolais animals; this confirms the findings of Allais et al. [36], who reported the highest frequency for the Q204X mutation in the Charolais breed. Other causal mutations in the *MSTN* gene that vary in frequency among breeds (e.g., [31, 32, 35, 36] have also been described. Among them, the F94L mutation, located 2 kb upstream of the Q204X mutation and extensively characterized in the Limousine breed [32], exhibited significant effects. However, it did not rank among the most significant variants within the region in either the within-breed GWAS or the MA conducted in this study, probably because it was poorly imputed in the Limousine breed. In the vicinity of the *MSTN* gene, lead variants of certain QTL were located in other positional candidate genes, with *HS6ST1*, *HERC2*, *WDR75*, *COL3A1*, and *ANKAR* being of particular interest. The *heparan sulfate 6-O-sulfotransferase 1* (*HS6ST1*) gene has previously been identified as a good functional candidate gene for fatty acid composition in muscle of crossbred Wagyu × Limousine animals [37]. The *HECT and RLD domain containing E3 ubiquitin protein ligase 2* (*HERC2*) gene was found to be associated with calving performance in Charolais cows [38] while the *WD repeat domain 75* (*WDR75*), *collagen type III alpha 1 chain* (*COL3A1*), and *ankyrin and armadillo repeat containing* (*ANKAR*) genes have been all reported as candidate genes for skeletal and muscularity type traits in Charolais and Limousine animals [28, 39].

On BTA6, within-population GWAS highlighted lead variants within the intergenic region between the *LCORL* and *SLIT2* genes, whereas in the meta-analyses these variants were directly located in *LCORL*, suggesting that this could be the causal gene responsible for the effects observed in this region. The *LCORL* gene encodes a transcription factor (ligand-dependent nuclear receptor corepressor-like) with a potential function in spermatogenesis. Variants in this gene have been found to be associated with stature in humans [40] and in cattle [5], as well as with other growth traits in cattle [25–27]. In the present study, the lead variants of the QTL with the most significant effects were intronic (rs381823183, rs109114124, rs109732906, rs110995268, rs109188585, and rs109256415), missense (rs109696064), and frameshift (rs384548488). Both the intronic rs110995268 and missense rs109696064 variants were also highlighted as possible causal variants by Wang et al. [26] for carcass traits. With a SIFT score of 0.4, the rs109696064 missense variant is predicted to be tolerated. Although Lindholm-Perry et al. [41] found correlations between the abundance of *LCORL* transcripts in muscle/adipose tissues and average daily gain and feed intake, the functional mechanisms linking this gene to growth traits remain unknown. Very close to *LCORL*, the *NCAPG* gene has often been reported as a candidate gene for growth traits [42, 43], but none of the lead variants of the QTL identified in the present meta-analyses were located in this gene. In the same region, other genes have also been proposed as positional candidate genes, such as the *mediator complex subunit 28* (*MED28*) [27] and *slit guidance ligand 2* (*SLIT2*) genes [28]. Interestingly, among the variants with significant effects that we detected here, some were reported to regulate the expression of *MED28* or *SLIT2* in CattleGTEx [23]. The *SLIT2* gene has also been reported to be associated with skeletal type traits in Angus and Limousine breeds [28]. In the same region, the *coiled-coil serine-rich protein 1* (*CCSER1*) and *potassium voltage-gated channel interacting protein 4* (*KCNIP4*) genes, which were found among the best candidates in the present study, were also reported to be associated with skeletal type traits in Angus, Charolais, or Limousine breeds [28].

In addition to the genes described above on BTA2 and 6, our meta-analyses led to the identification of other positional and functional candidate genes. For example, for all the QTL detected on BTA14 by the meta-analyses, the lead variant was located in the *pleomorphic adenoma gene 1* (*PLAG1*) gene, which encodes a transcription factor that after activation results in the upregulation of target genes such as *IGF2*, which encodes a polypeptide growth factor involved in development and growth. This gene has been associated with stature and other growth and morphology traits in many studies conducted on cattle, e.g., [5, 25, 26, 39, 44, 45]. In a large-scale GWAS meta-analysis conducted on WGS data as part of the 1000 Bull Genomes project, the variant with the most significant effects on cattle stature (rs109815800) was located in an intron of the *PLAG1* gene [5]. This variant was also found to be the lead variant in two of the meta-analyses we conducted, in spite of the fact that it was never the variant with the most significant effects in any of the within-population GWAS. Here, we also identified two other variants located in the 5′UTR (rs210030313) and downstream regions (rs134215421) of this gene. Interestingly, rs210030313, which was identified as a causal candidate variant for cattle stature by Karim et al. [44], was predicted to be located in a transcriptional binding site [46], suggesting that expression of the PLAG1 transcription factor might itself be regulated by another transcription factor. Instead, the rs134215421 variant, located 1166 bp downstream of *PLAG1*, was identified as the lead variant for average daily gain and metabolic body weight by Zhang et al. [25].

## Conclusions

Compared to within-population GWAS, large-scale meta-analyses conducted at the sequence level, coupled with post-GWAS analyses, significantly improved the identification of genes and candidate causal variants associated with beef production traits in cattle. Our study also highlights the usefulness of searching for expression and splicing quantitative trait loci (e/sQTL) that overlap with QTL, as this can help identify new candidate genes and prioritize candidate variants in the QTL regions. By shedding light on the biological mechanisms underlying these traits, an approach that combines meta-analyses with post-GWAS analyses has the potential to facilitate the direct selection of favorable causal alleles.

### Supplementary Information

**Additional file 1: Table S1.** QTL detected in within-population GWAS and fixed-effects and z-score meta-analyses.

**Additional file 2: Figure S1.** Manhattan plots of each meta-analysis from within-population GWAS and MA results.

**Additional file 3: Table S2.** Results of QTL annotation and enrichment analyses in within-population GWAS and MA.

## Data Availability

The genotypes and phenotypes used in the GWAS conducted by each partner are not publicly accessible. However, they can be obtained from the authors upon reasonable request following execution of a material transfer agreement, and with the permission of the corresponding partner.
