# Correction: Sequence-based GWAS meta-analyses for beef production traits

**DOI:** 10.1186/s12711-023-00852-9

**Published:** 2023-11-13

**Authors:** Marie-Pierre Sanchez, Thierry Tribout, Naveen K. Kadri, Praveen K. Chitneedi, Steffen Maak, Chris Hozé, Mekki Boussaha, Pascal Croiseau, Romain Philippe, Mirjam Spengeler, Christa Kühn, Yining Wang, Changxi Li, Graham Plastow, Hubert Pausch, Didier Boichard

**Affiliations:** 1 Université Paris-Saclay, INRAE, AgroParisTech, GABI, 78350 Jouy-en-Josas, France; 2 Animal Genomics, ETH Zurich, 8092 Zurich, Switzerland; 3 Research Institute for Farm Animal Biology (FBN), 18196 Dummerstorf, Germany; 4 Eliance, 75595 Paris, France; 5 INRAE, USC1061 GAMAA, Université de Limoges, 87060 Limoges, France; 6 QualitasAG, 6300 Zug, Switzerland; 7 Agricultural and Environmental Faculty, University Rostock, 18059 Rostock, Germany; 8 Present Address: Friedrich-Loefer-Institut (FLI), Insel Riems, 17493 Greifswald, Germany; 9 Lacombe Research and Development Centre, Agriculture and Agri-Food Canada, Lacombe, AB T4L 1W1 Canada; 10 Department of Agricultural, Food and Nutritional Science, Livestock Gentec, University of Alberta, Edmonton, AB T6G 2HI Canada

**Correction: Genetics Selection Evolution (2023) 55:70** 10.1186/s12711-023-00848-5

Following publication of the original article [1], it has been reported that the incorrect copyright holder was used. The correct copyright holder is: © His Majesty the King in Right of Canada as represented by the Minister of Agriculture and Agri-Food Canada. The original article [1] has been corrected.
