# Supplementary material for: Sequence-based GWAS meta-analyses for beef production traits
Source: Genet Sel Evol. 2023 Oct 12;55:70. doi: 10.1186/s12711-023-00848-5 (PMC10568825; doi:10.1186/s12711-023-00848-5)

G1 meta-analysis

Partner FBN - Population HC_CALVES - Trait BW

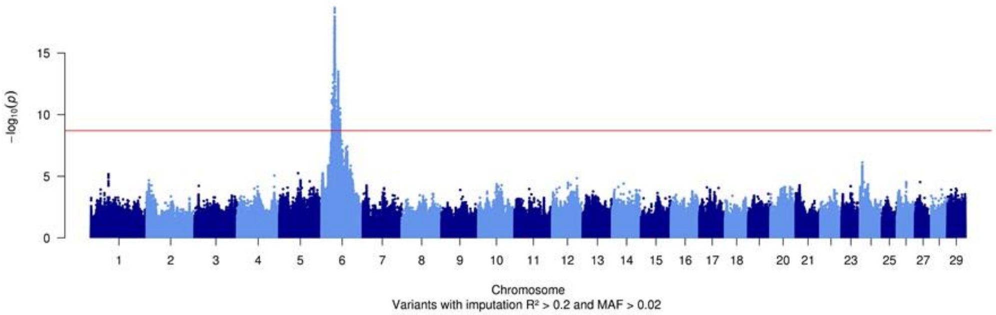

Partner INRAE - Population LIM_STEERS - Trait BW

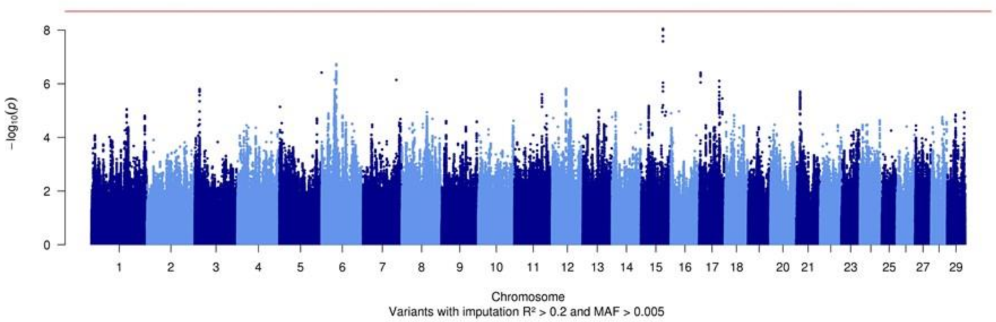

Partner INRAE - Population CHA_STEERS - Trait BW

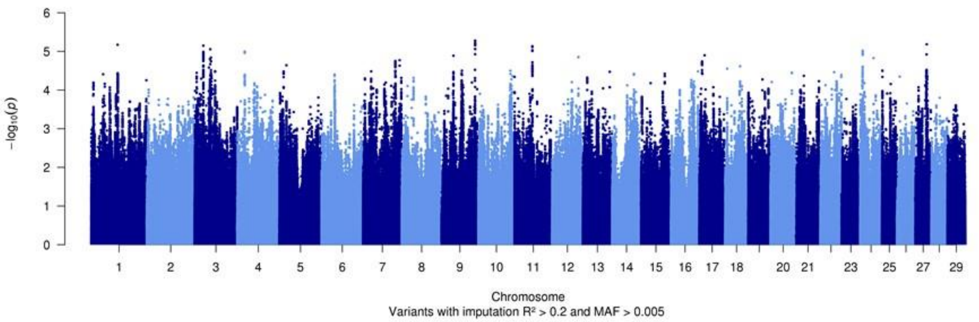

fixed effects meta-analysis method with 3 within-population GWAS

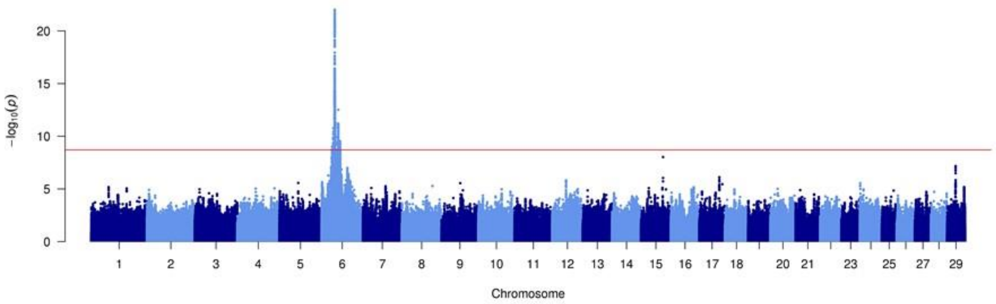

z-score meta-analysis method with 3 within-population GWAS

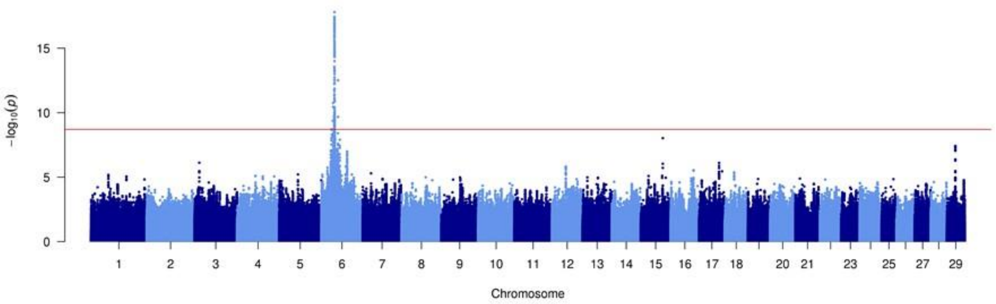

G2 meta-analysis

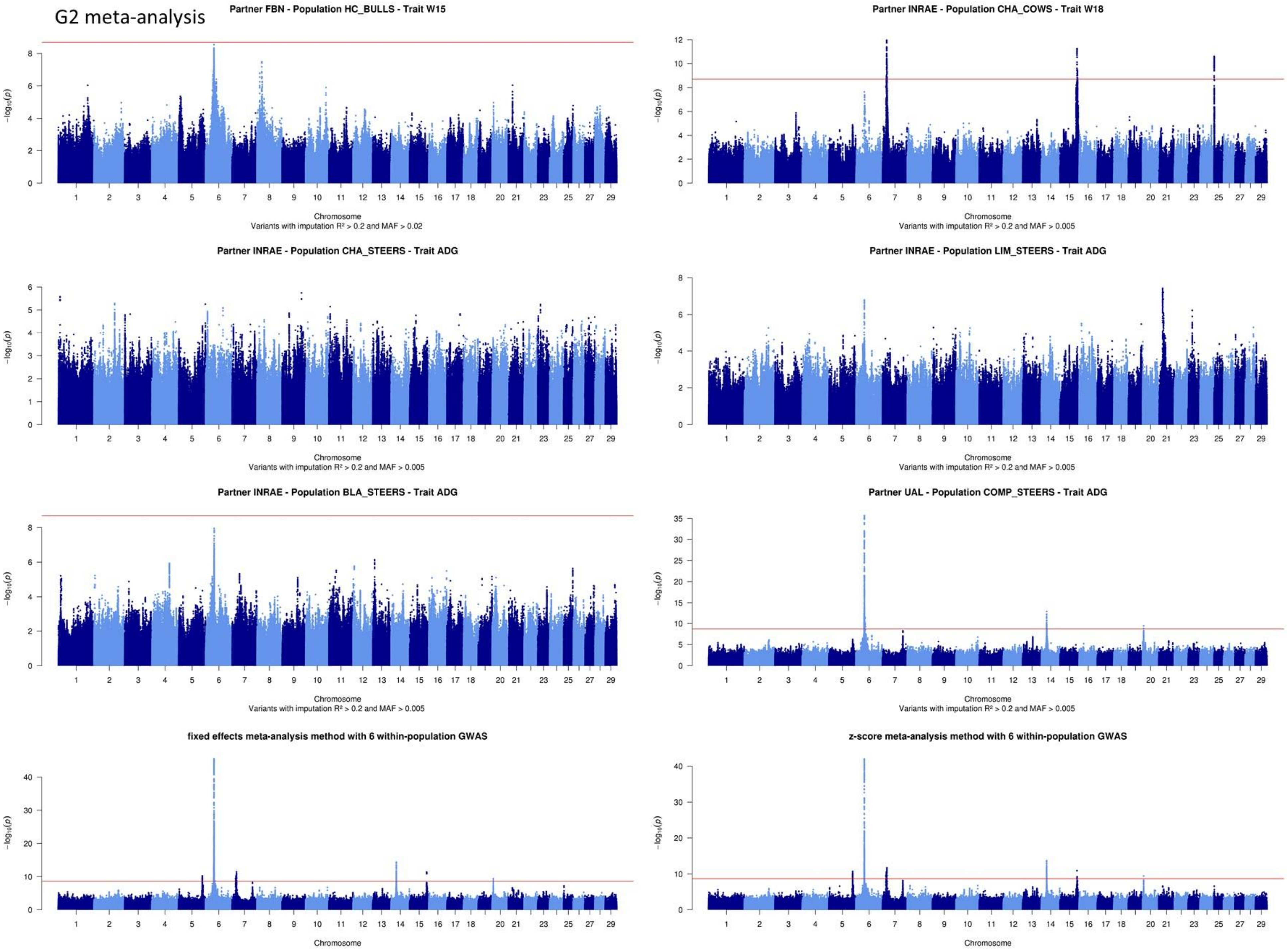

G3 meta-analysis

Partner INRAE - Population BLA_STEERS - Trait WS

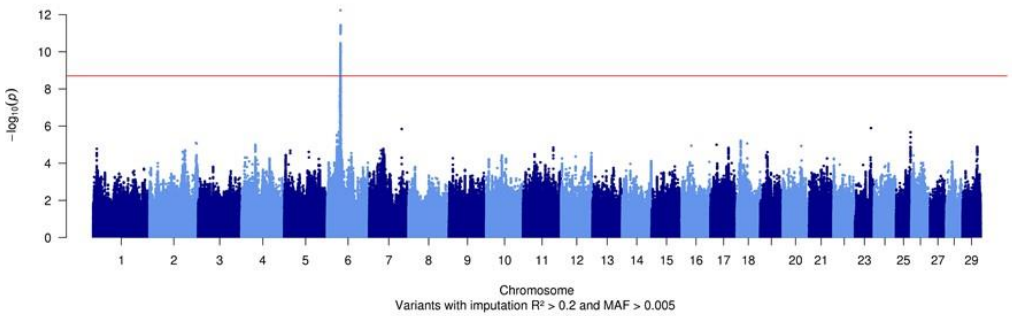

Partner FBN - Population HC_BULLS - Trait WS

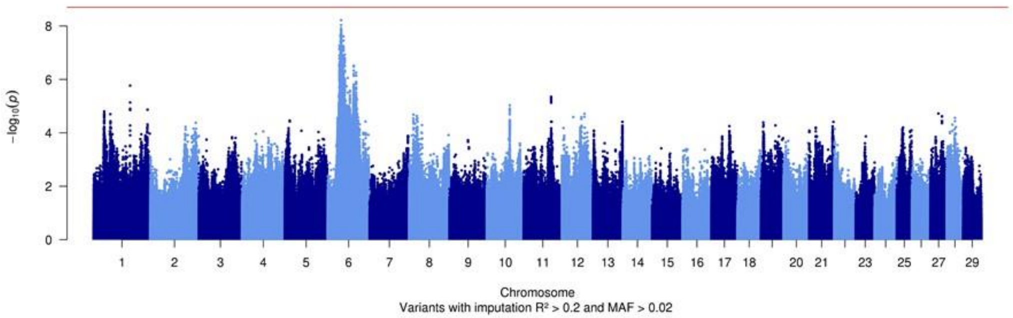

Partner INRAE - Population LIM_STEERS - Trait WS

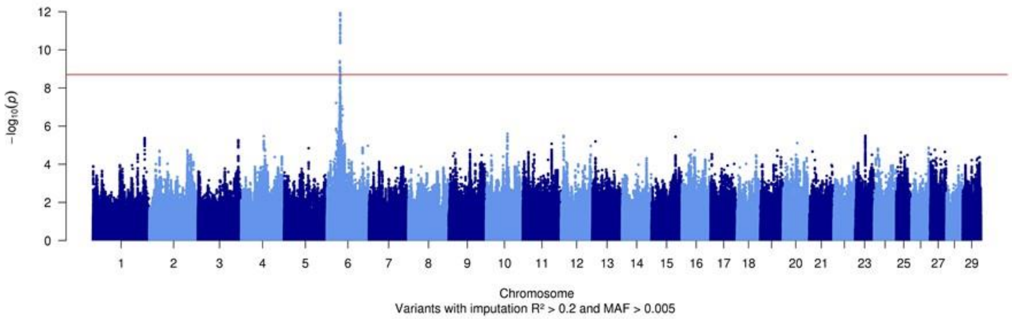

Partner FBN - Population HC_COWS - Trait WS

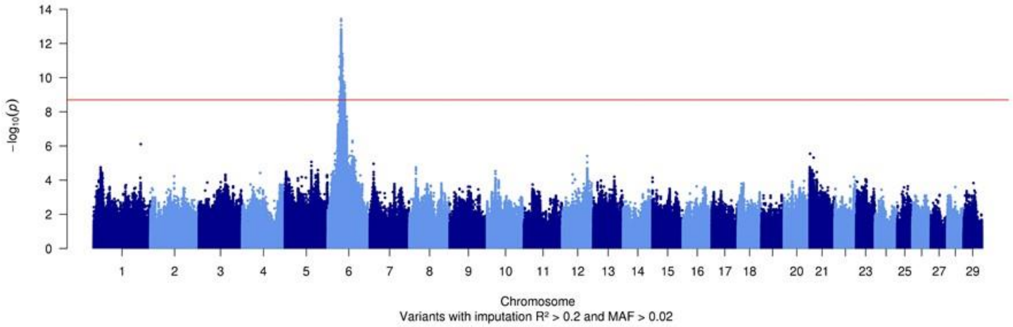

fixed effects meta-analysis method with 4 within-population GWAS

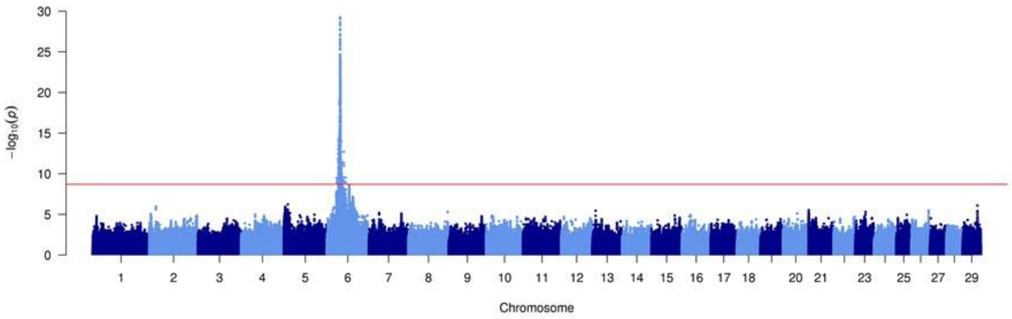

z-score meta-analysis method with 4 within-population GWAS

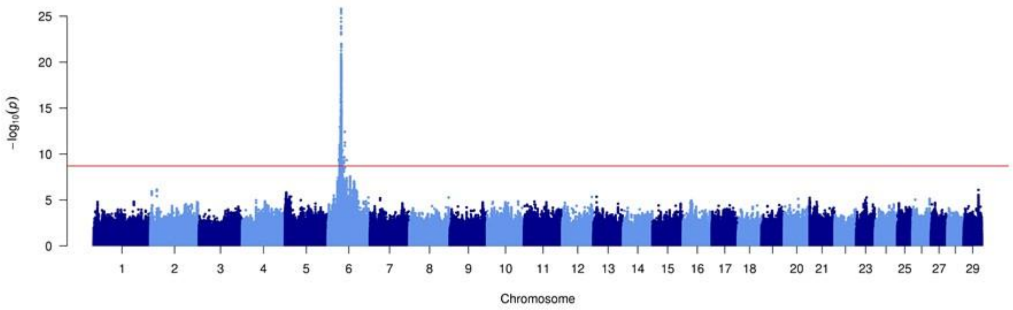

M1 meta-analysis

Partner INRAE - Population BLA_STEERS - Trait MD

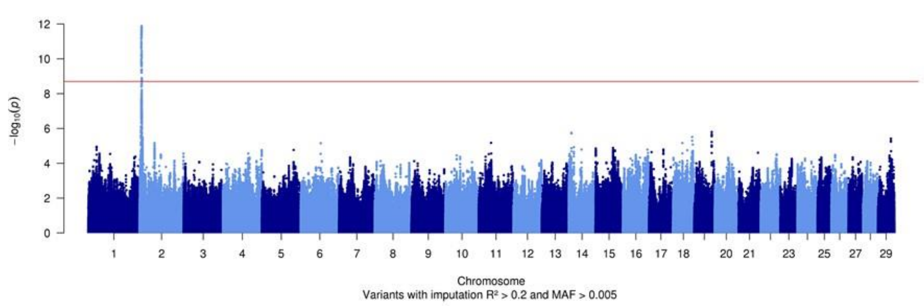

Partner INRAE - Population CHA_COWS - Trait MS30

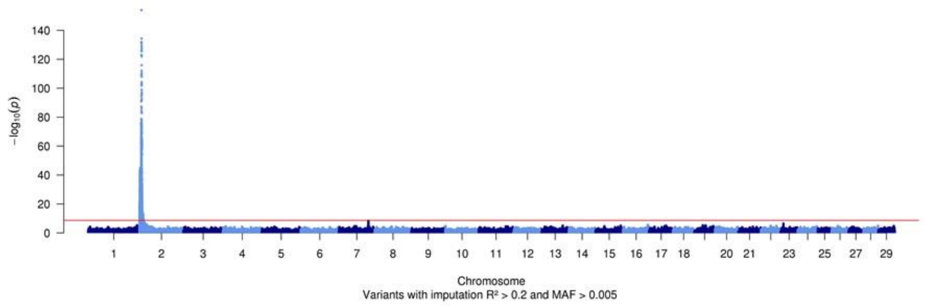

Partner INRAE - Population LIM_STEERS - Trait MD

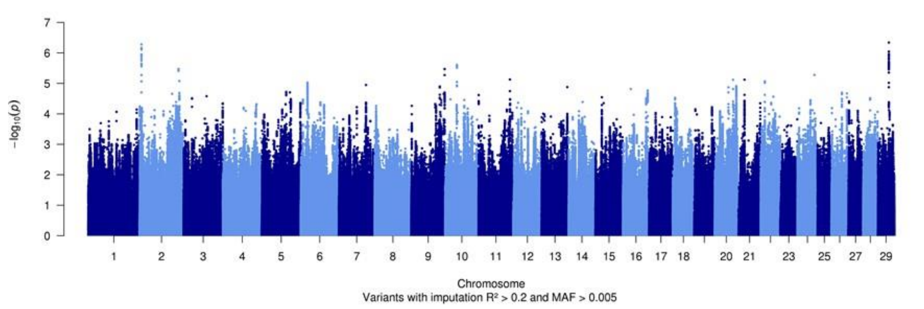

Partner INRAE - Population CHA_STEERS - Trait MD

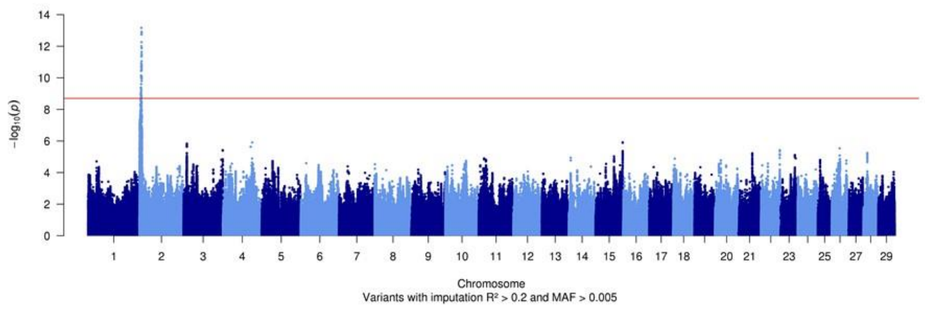

Partner INRAE - Population MON_BULLS - Trait THIGHS

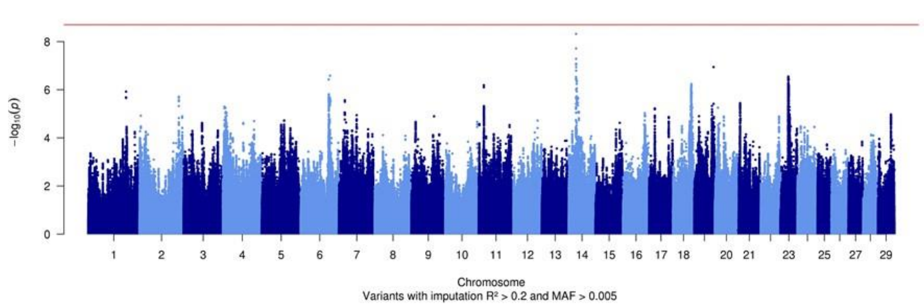

Partner INRAE - Population NOR_BULLS - Trait THIGHS

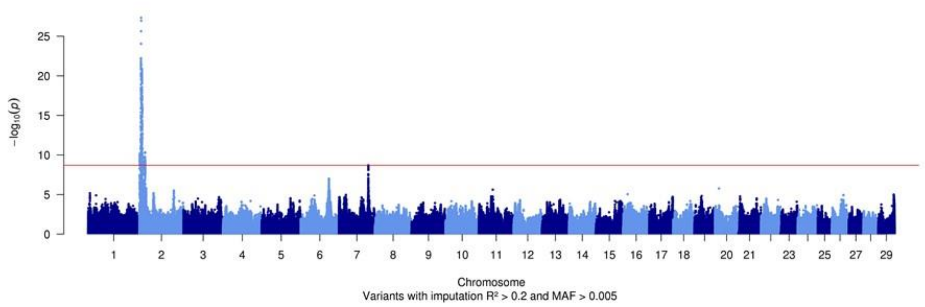

fixed effects meta-analysis method with 6 within-population GWAS

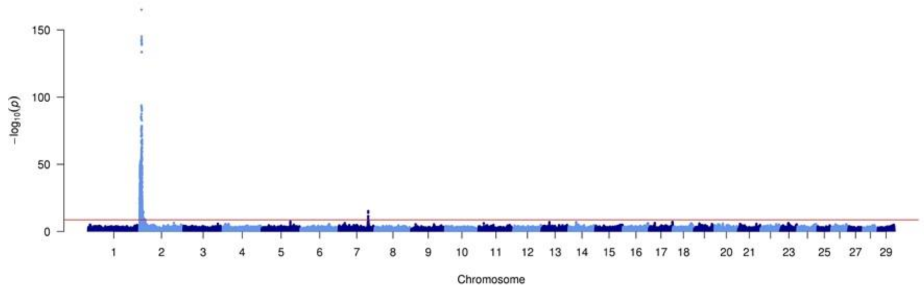

z-score meta-analysis method with 6 within-population GWAS

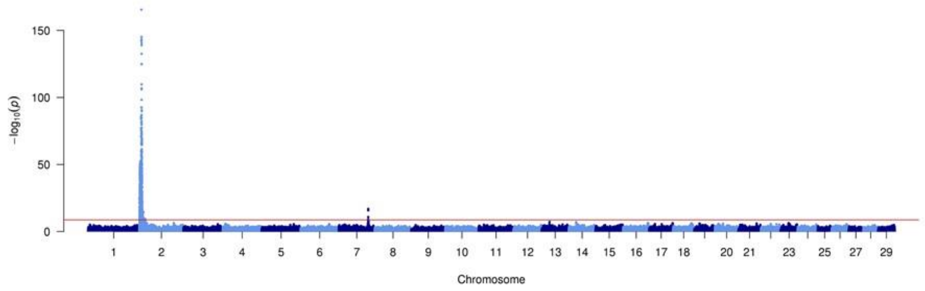

M2 meta-analysis

Partner INRAE - Population CHA_STEERS - Trait MD

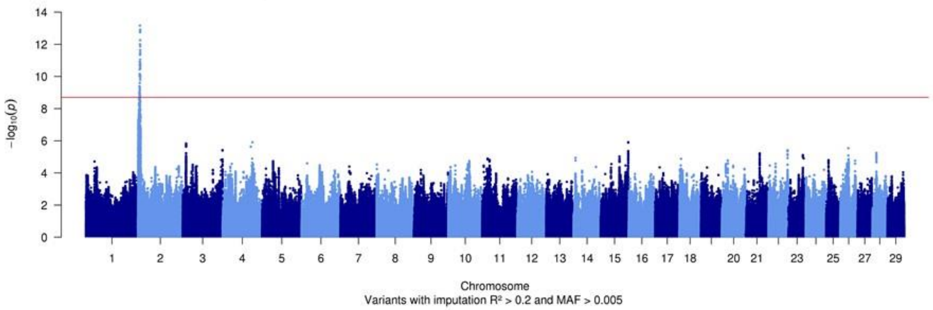

Partner INRAE - Population CHA_COWS - Trait MS30

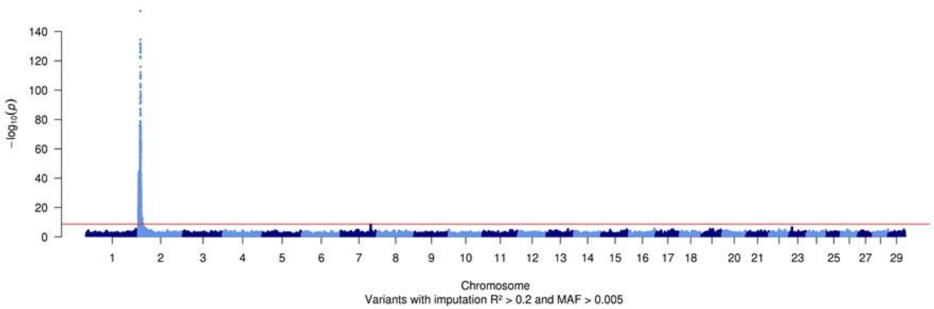

Partner INRAE - Population LIM_STEERS - Trait MD

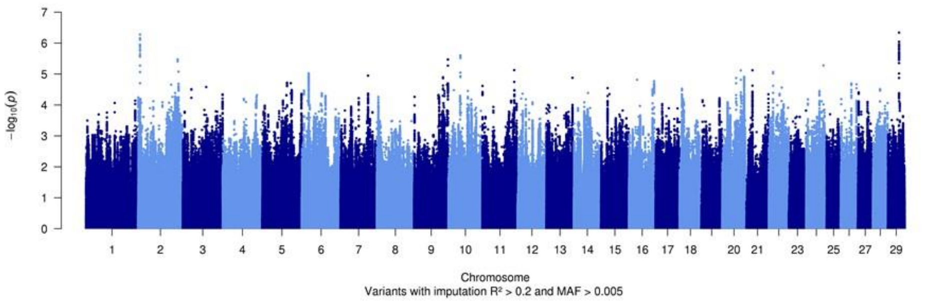

Partner INRAE - Population BLA_STEERS - Trait MD

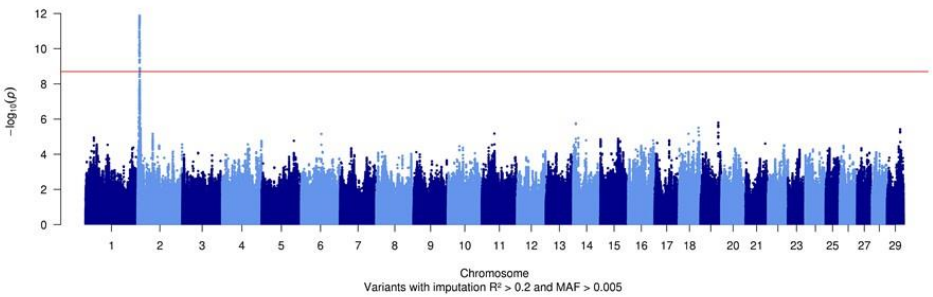

Partner INRAE - Population MON_BULLS - Trait WITHER

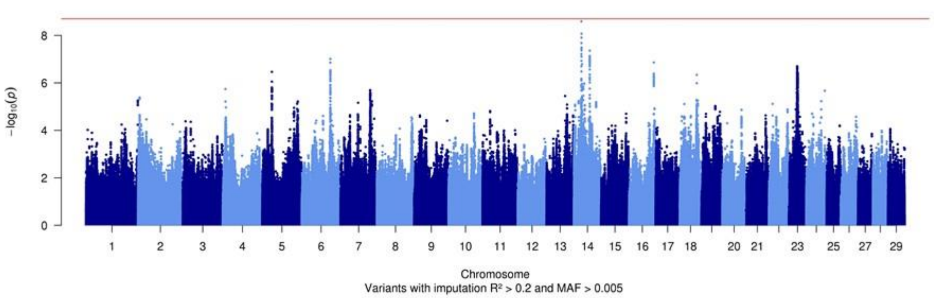

Partner INRAE - Population NOR_BULLS - Trait WITHER

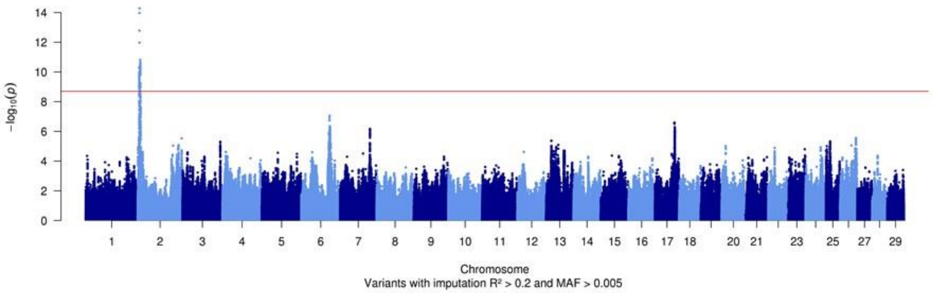

fixed effects meta-analysis method with 6 within-population GWAS

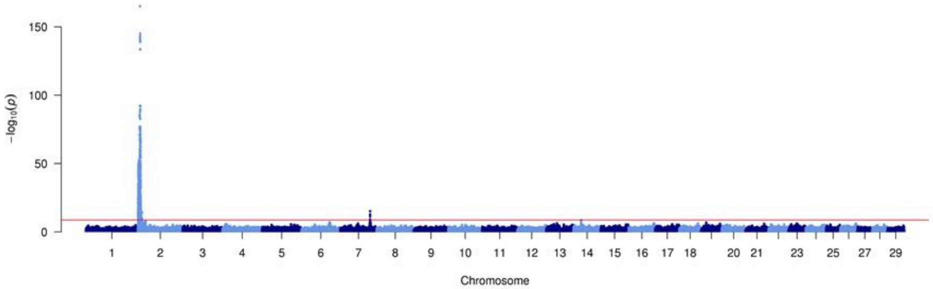

z-score meta-analysis method with 6 within-population GWAS

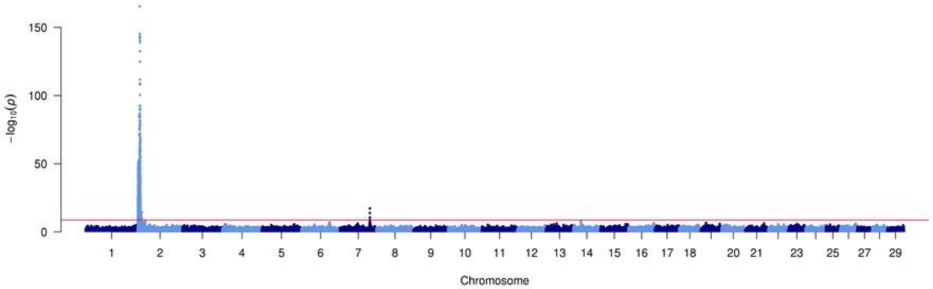

M3 meta-analysis

Partner FBN - Population HC_COWS - Trait LL

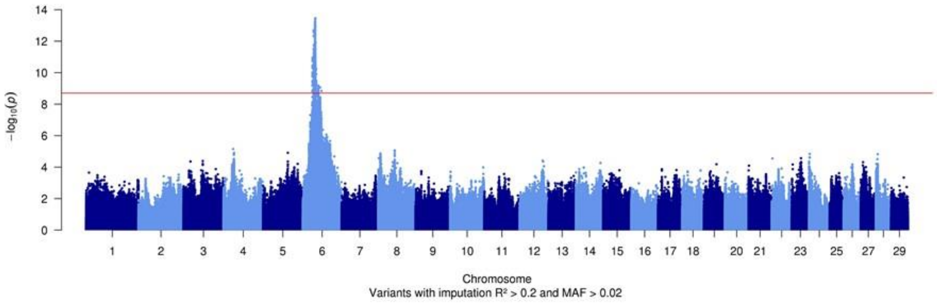

Partner FBN - Population HC_BULLS - Trait LL

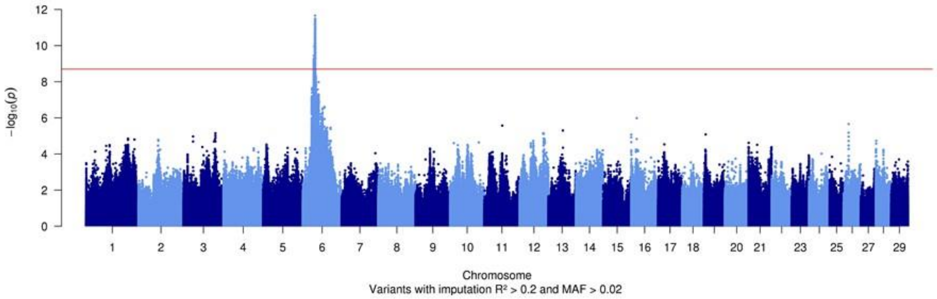

Partner INRAE - Population BLA_STEERS - Trait LL

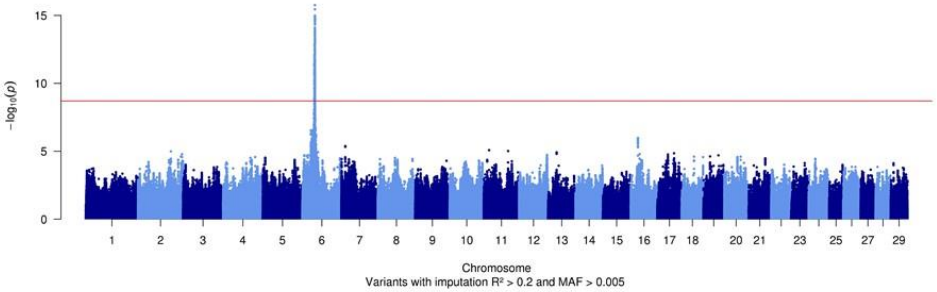

Partner INRAE - Population LIM_STEERS - Trait LL

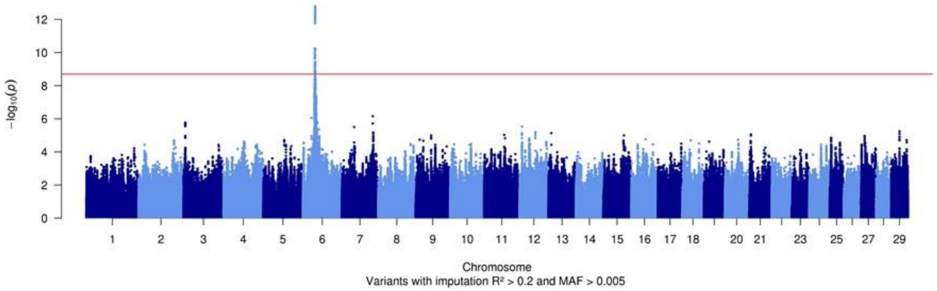

Partner INRAE - Population CHA_STEERS - Trait LL

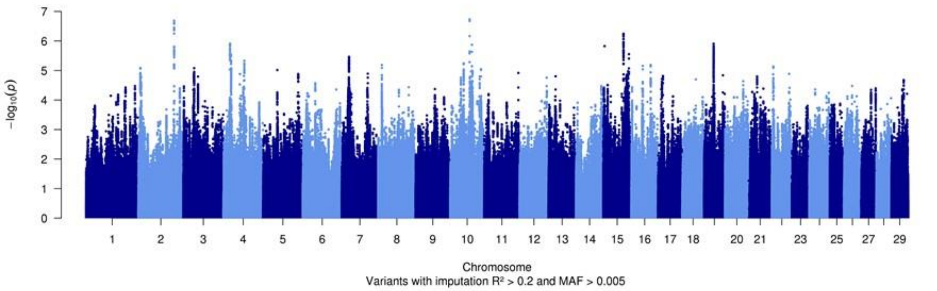

fixed effects meta-analysis method with 5 within-population GWAS

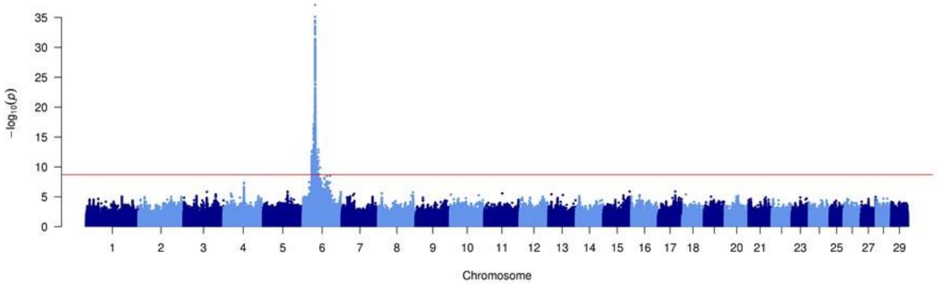

z-score meta-analysis method with 5 within-population GWAS

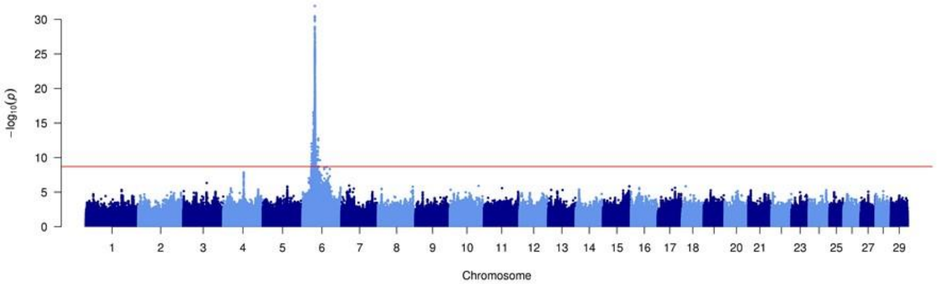

M4 meta-analysis

Partner FBN - Population HC_BULLS - Trait WT

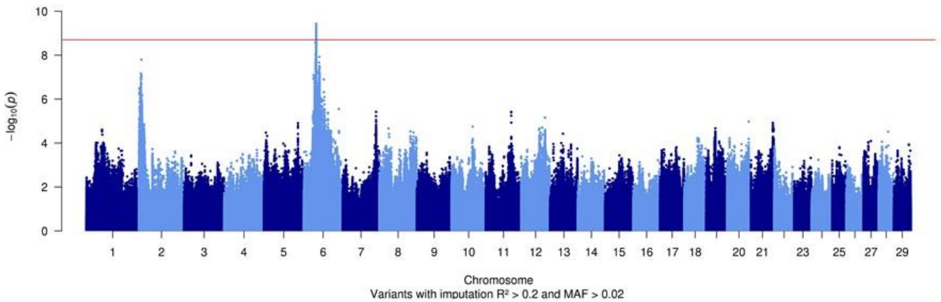

Partner FBN - Population HC_COWS - Trait WT

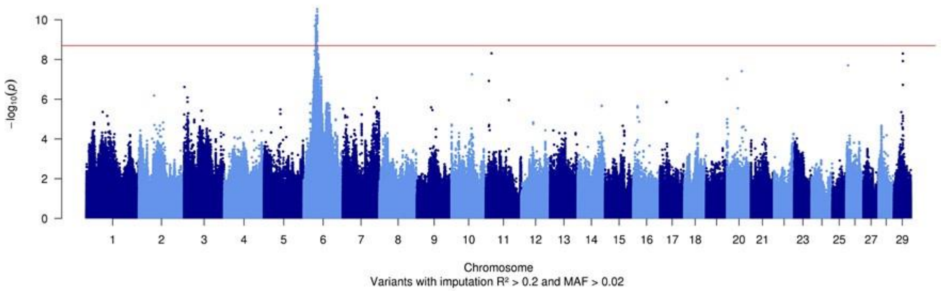

Partner INRAE - Population BLA_STEERS - Trait WT

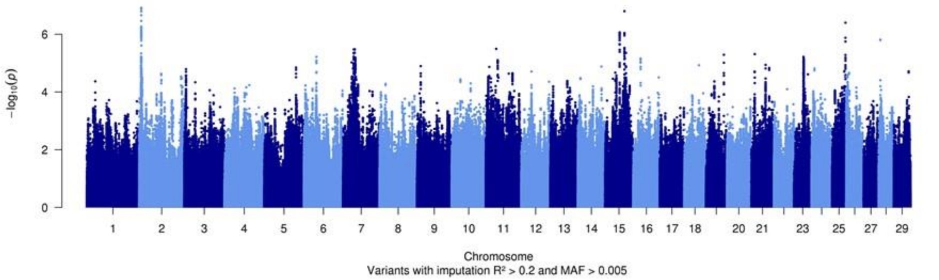

Partner INRAE - Population LIM_STEERS - Trait WT

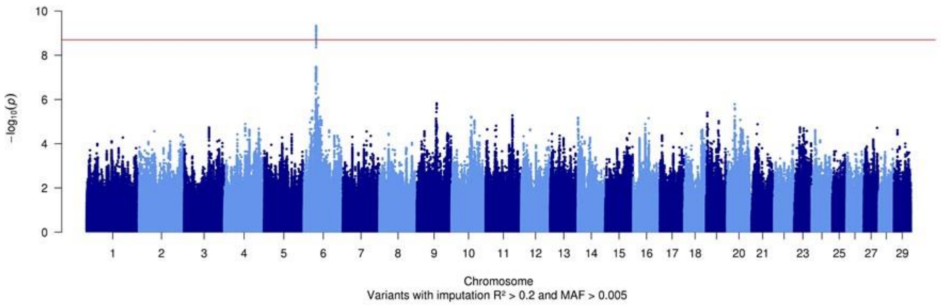

Partner INRAE - Population CHA_STEERS - Trait WT

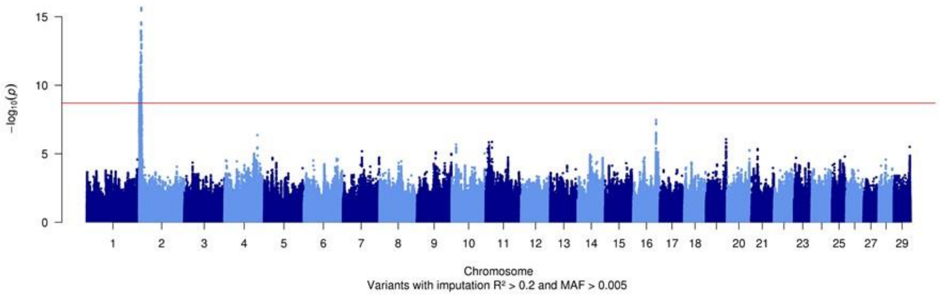

fixed effects meta-analysis method with 5 within-population GWAS

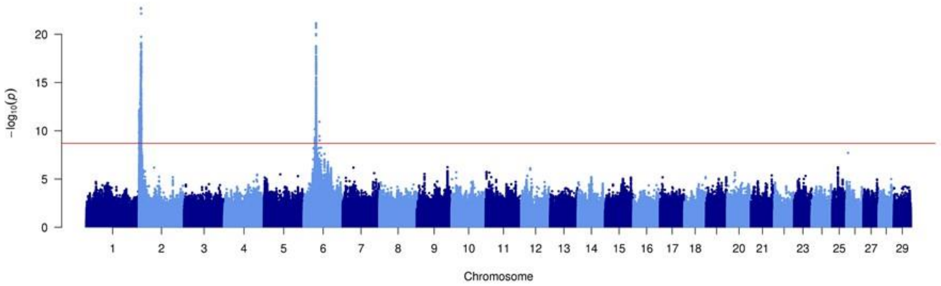

z-score meta-analysis method with 5 within-population GWAS

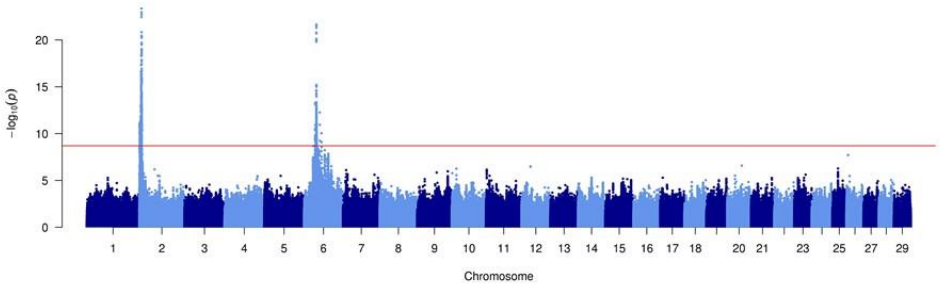

M5 meta-analysis

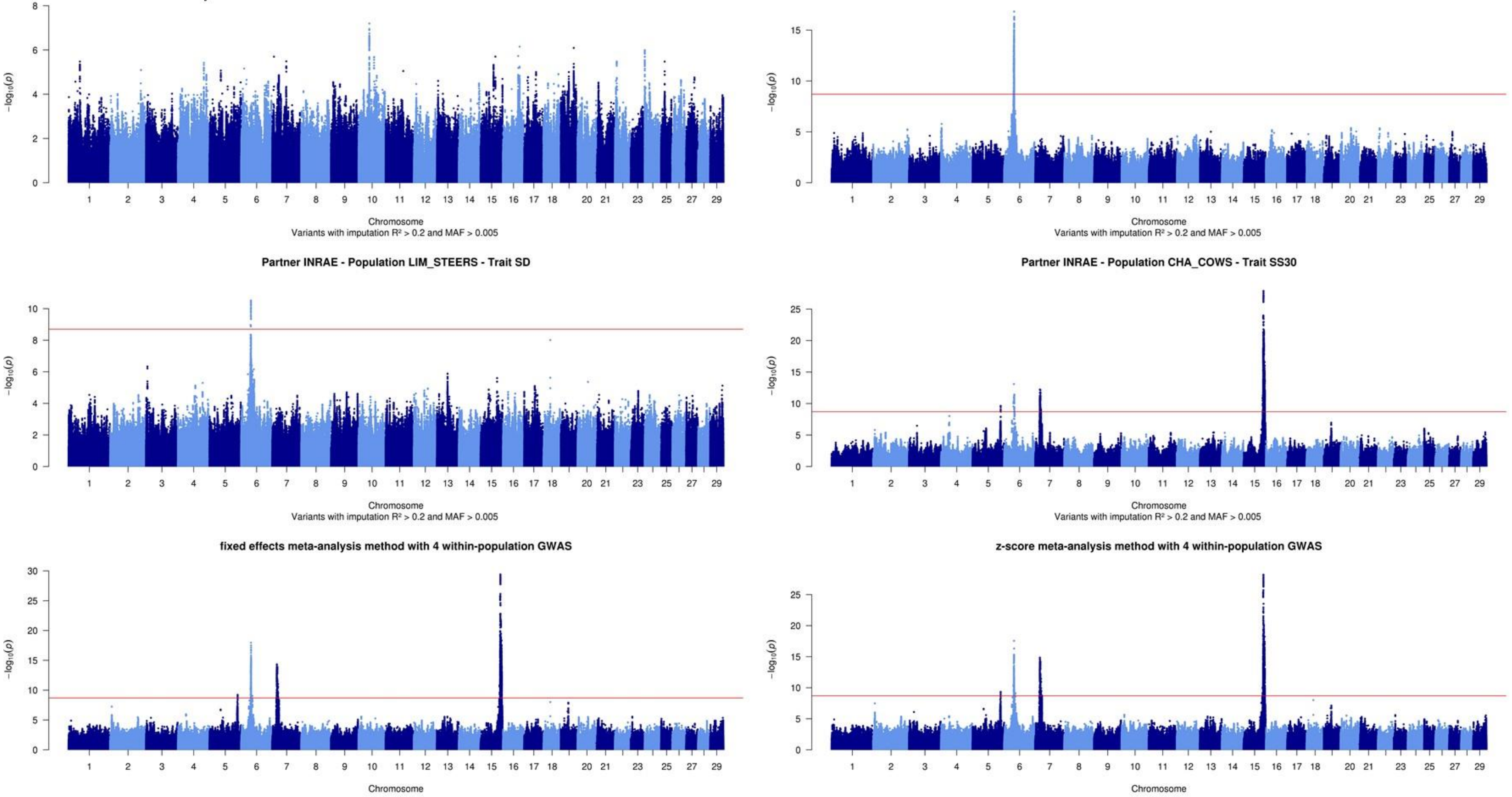

C1 meta-analysis

Partner FBN - Population HC_BULLS - Trait CW

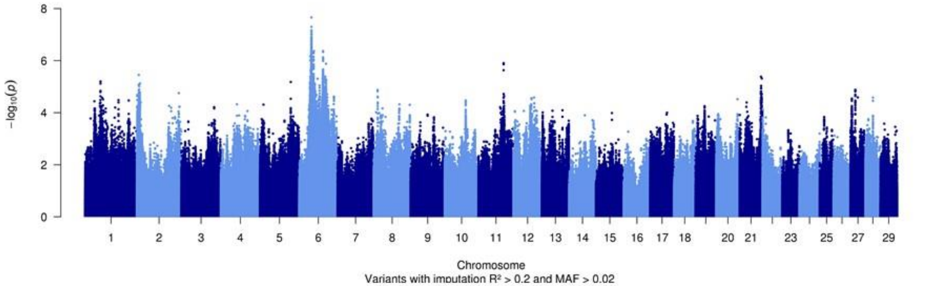

Partner INRAE - Population CHA_STEERS - Trait CW

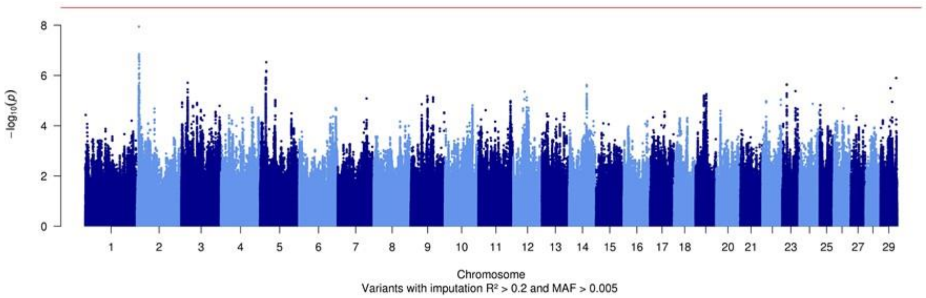

Partner FBN - Population HC_COWS - Trait CW

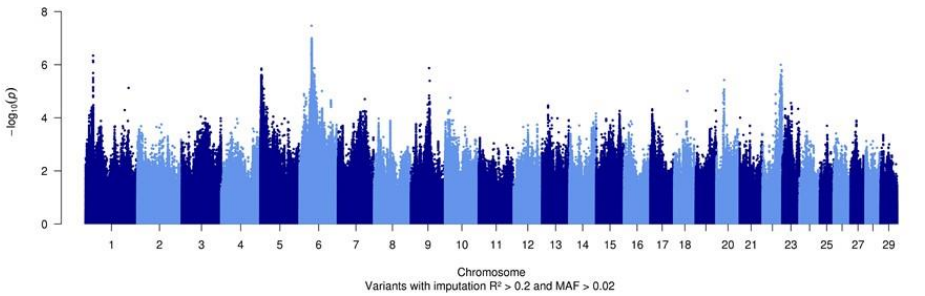

Partner INRAE - Population MON_STEERS - Trait CW

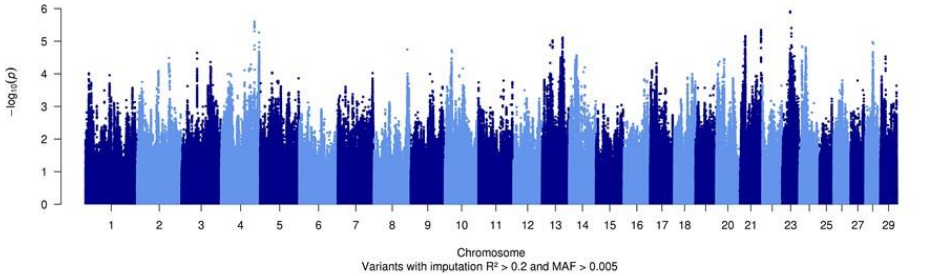

Partner ETH - Population BO_BULLS - Trait CW_B

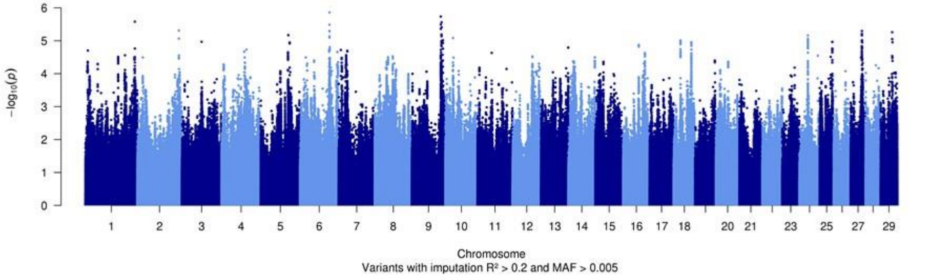

Partner INRAE - Population NOR_STEERS - Trait CW

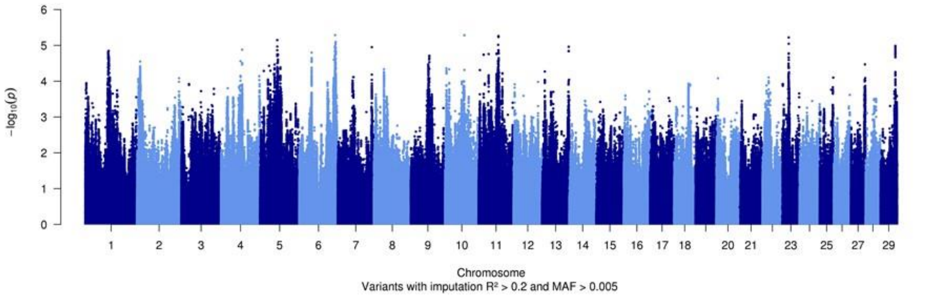

Partner UAL - Population COMP_STEERS - Trait HCW

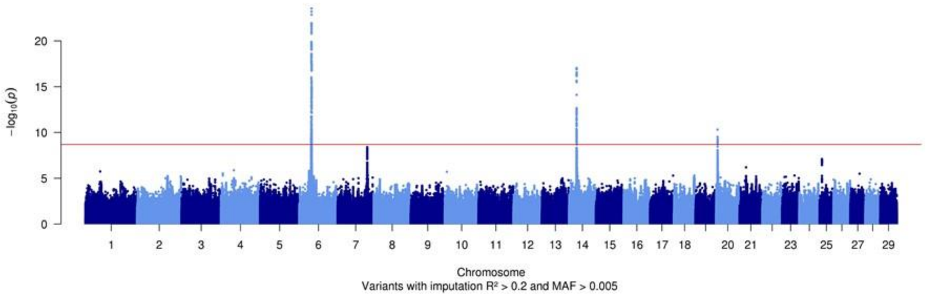

C2 meta-analysis

Partner INRAE - Population CHA_STEERS - Trait ASst

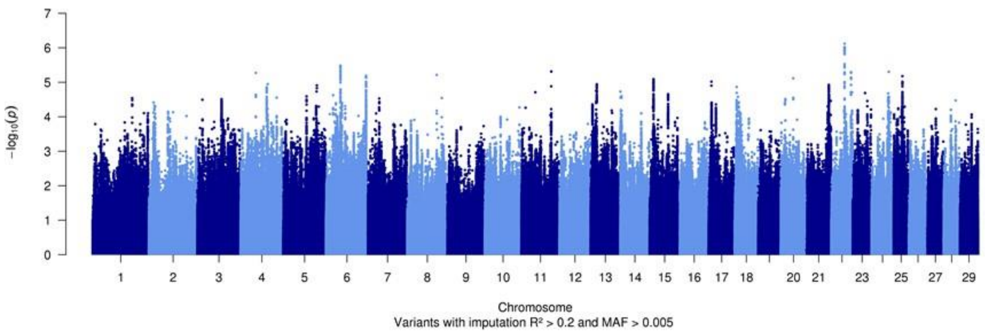

Partner INRAE - Population CHA_STEERS - Trait AS

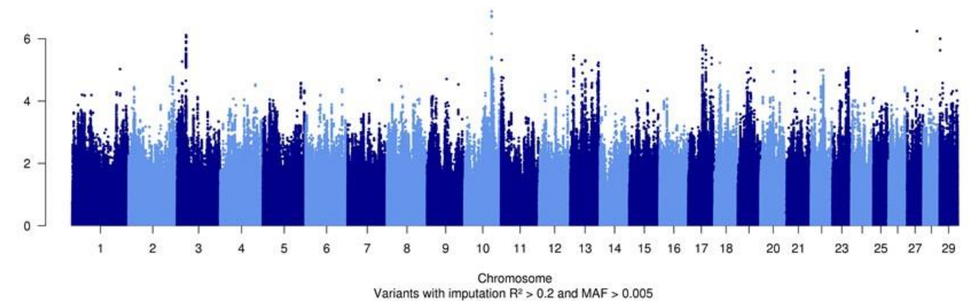

Partner INRAE - Population NOR_STEERS - Trait AS

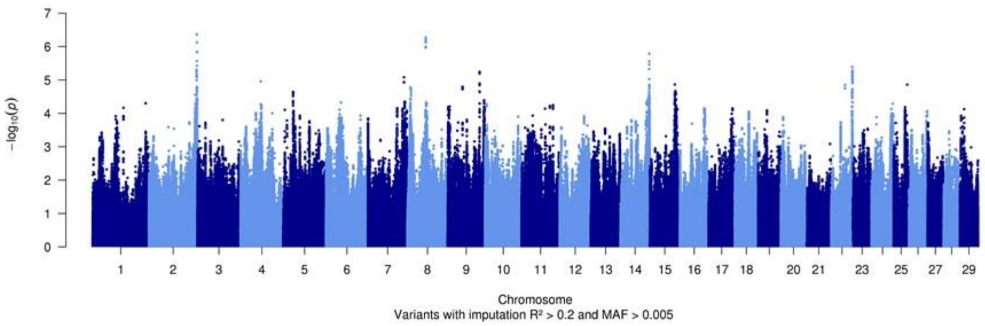

Partner INRAE - Population MON_STEERS - Trait AS

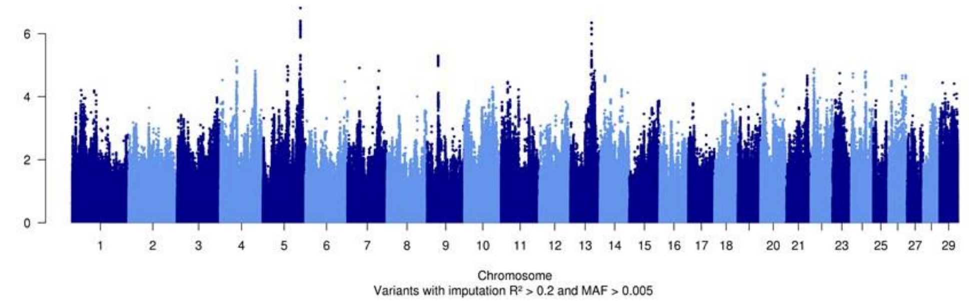

fixed effects meta-analysis method with 4 within-population GWAS

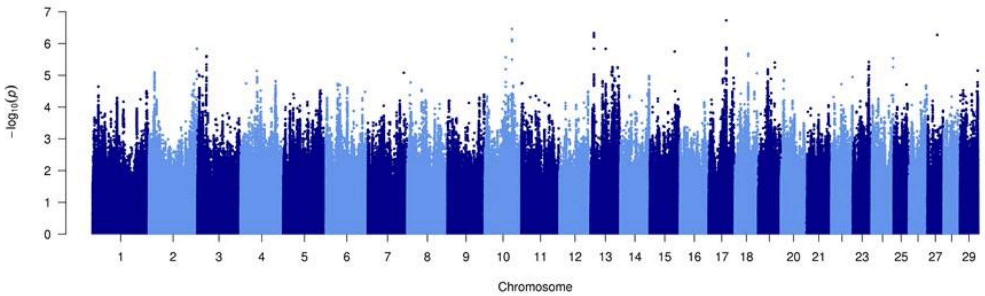

z-score meta-analysis method with 4 within-population GWAS

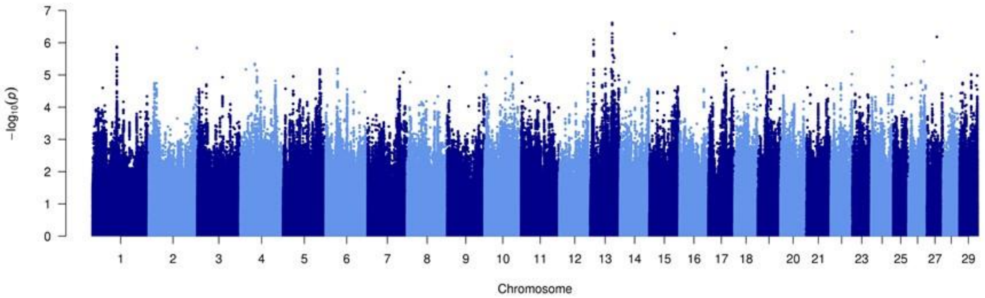

C3 meta-analysis

Partner FBN - Population HC_COWS - Trait CY

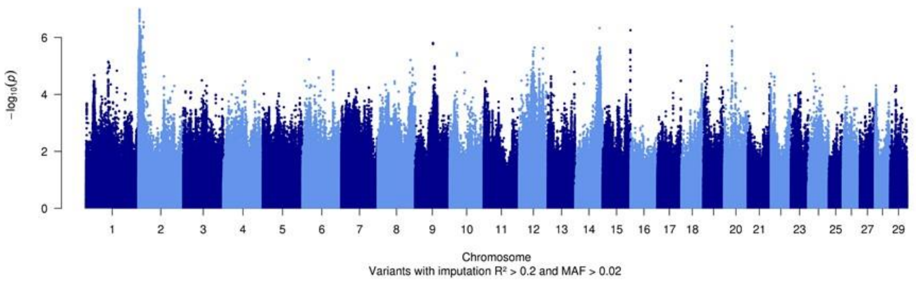

Partner INRAE - Population BLA_STEERS - Trait CY

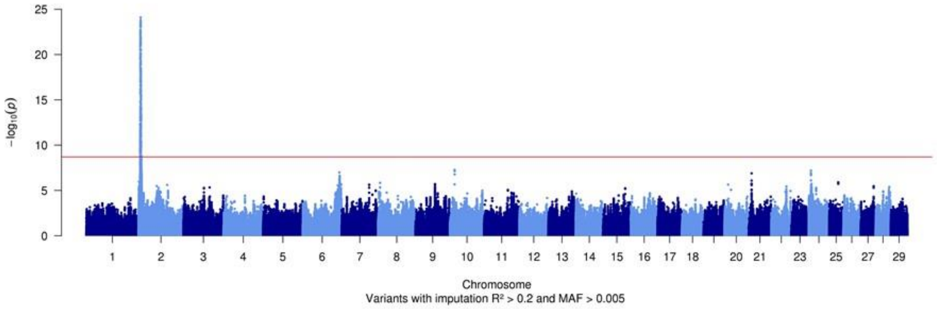

Partner FBN - Population HC_BULLS - Trait CY

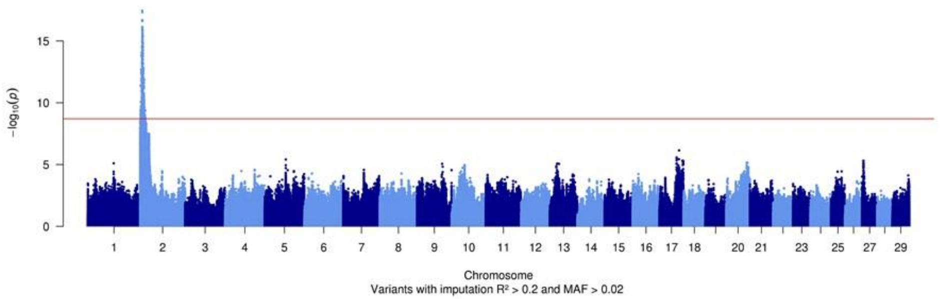

Partner INRAE - Population LIM_STEERS - Trait CY

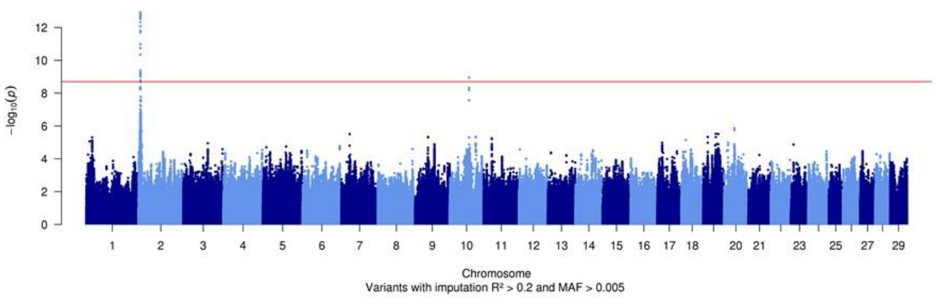

Partner INRAE - Population CHA_STEERS - Trait CY

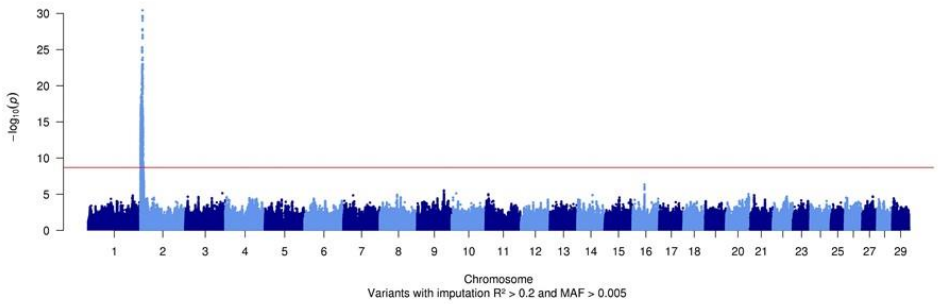

fixed effects meta-analysis method with 5 within-population GWAS

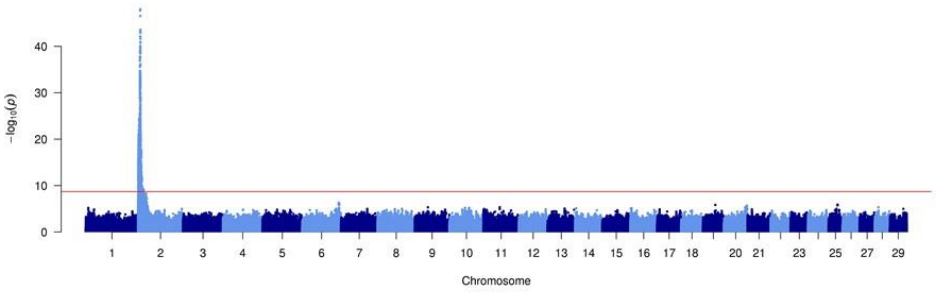

z-score meta-analysis method with 5 within-population GWAS

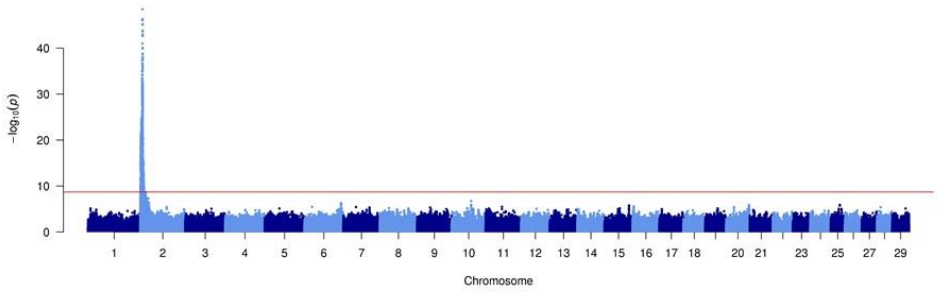

C4 meta-analysis

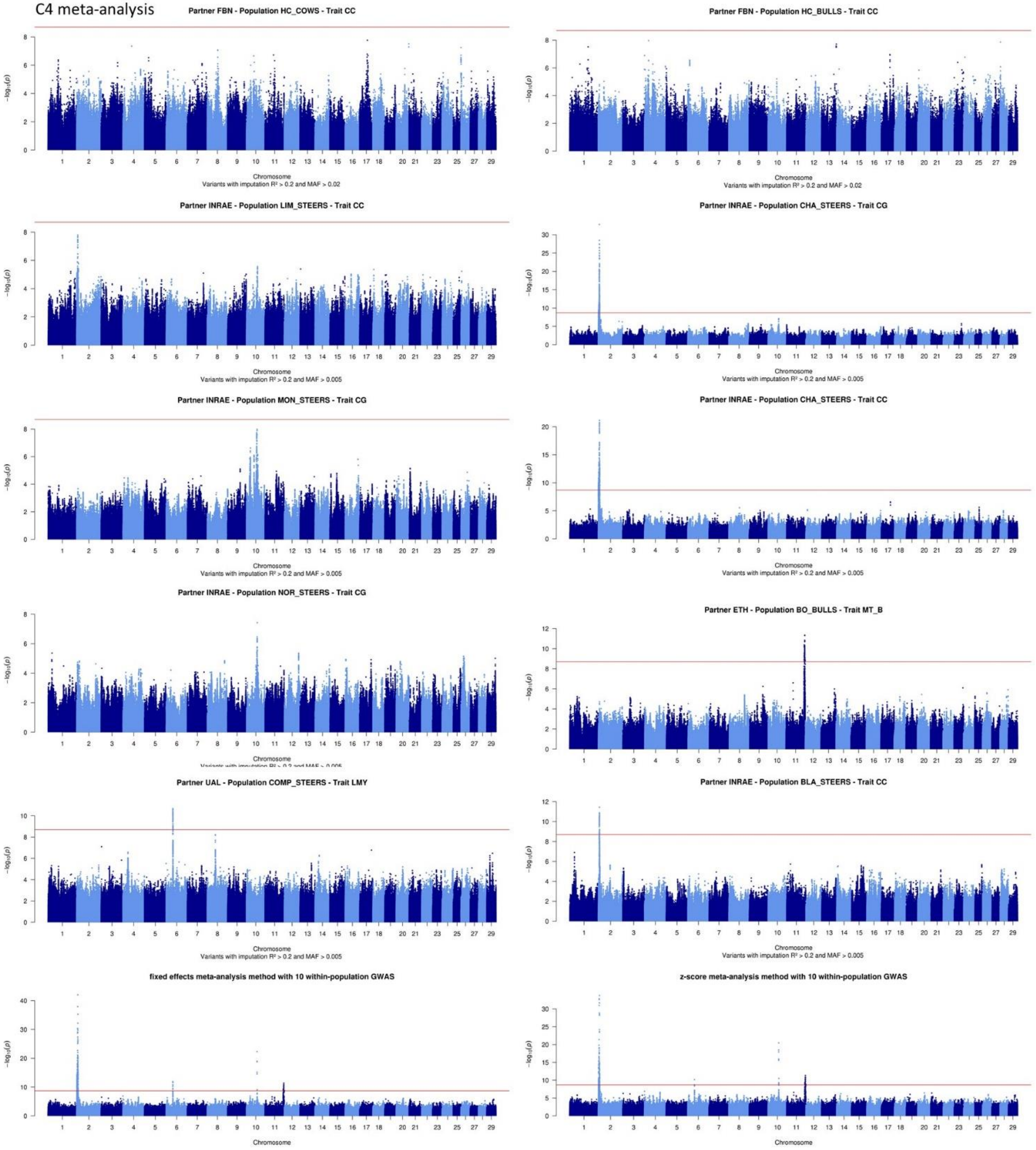

C5 meta-analysis

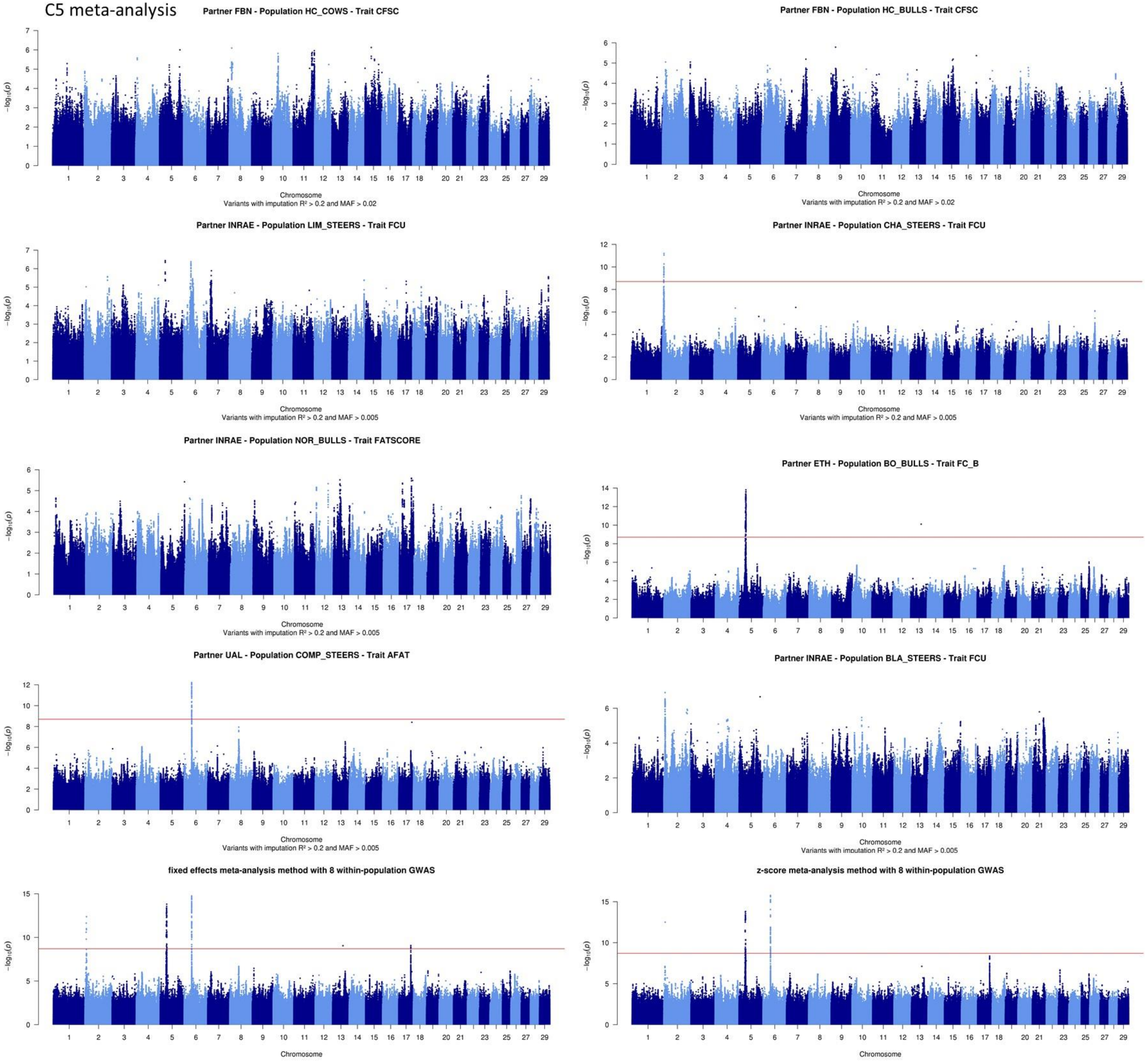

C6 meta-analysis

Partner FBN - Population HC_COWS - Trait ALT

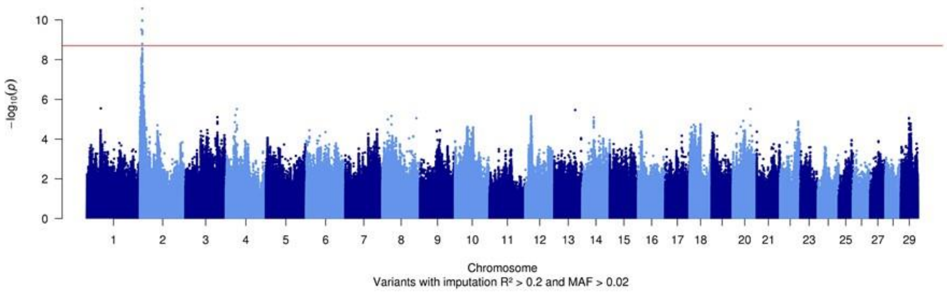

Partner INRAE - Population CHA_STEERS - Trait ALT

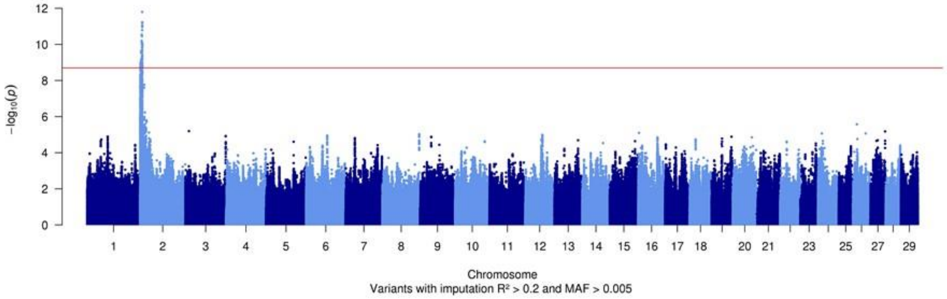

Partner FBN - Population HC_BULLS - Trait ALT

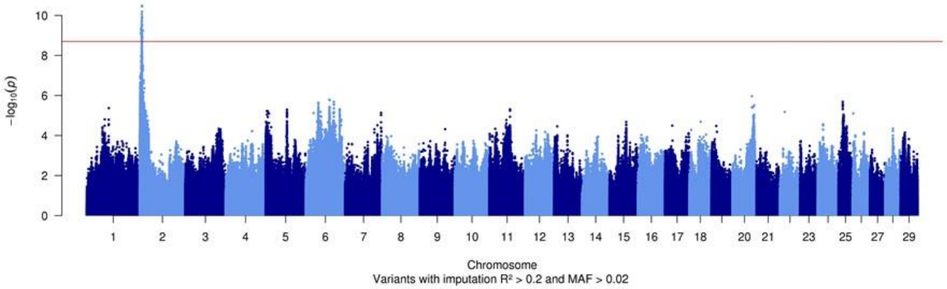

Partner INRAE - Population LIM_STEERS - Trait ALT

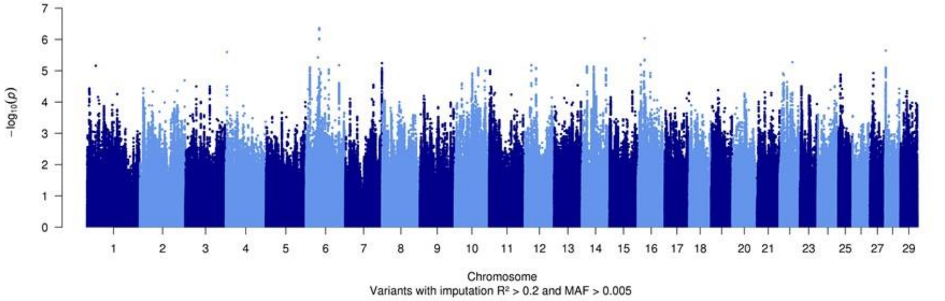

Partner INRAE - Population BLA_STEERS - Trait ALT

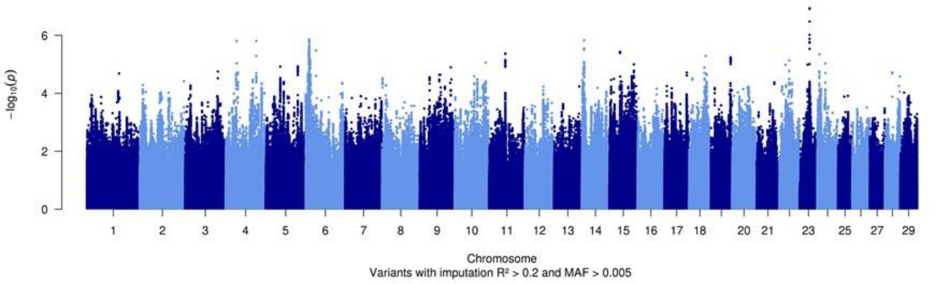

fixed effects meta-analysis method with 5 within-population GWAS

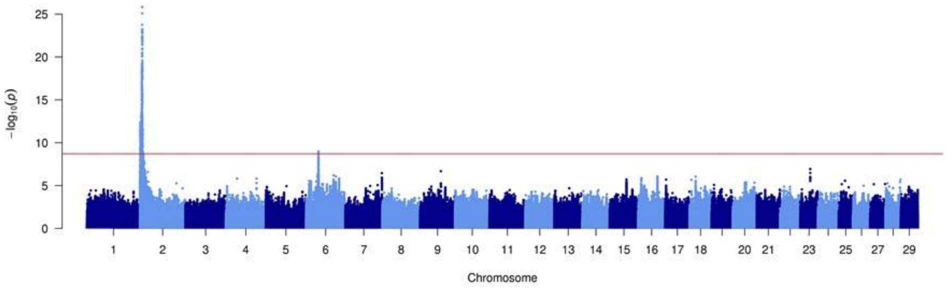

z-score meta-analysis method with 5 within-population GWAS

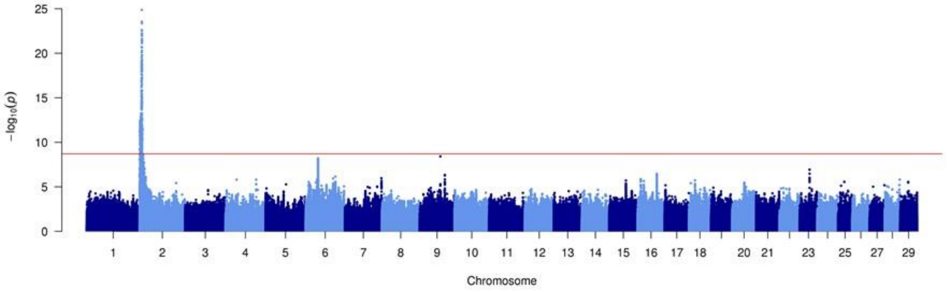

C7 meta-analysis

Partner FBN - Population HC_COWS - Trait IFW

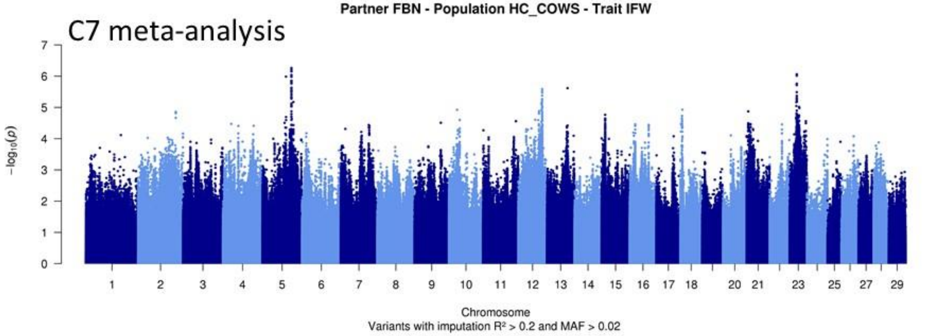

Partner INRAE - Population CHA_STEERS - Trait IFW

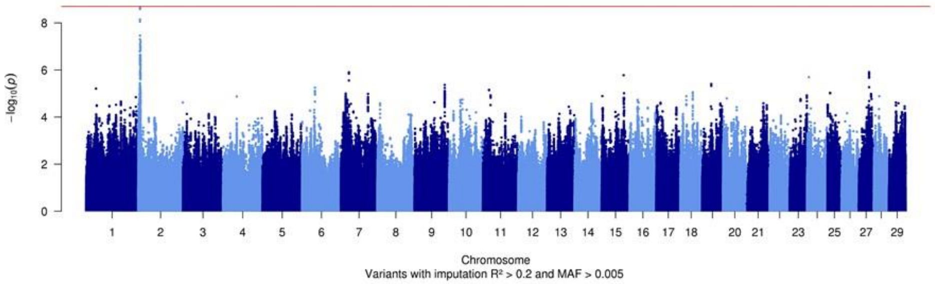

Partner FBN - Population HC_BULLS - Trait IFW

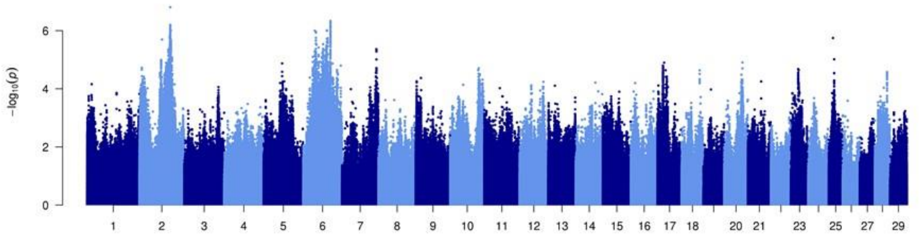

Partner INRAE - Population LIM_STEERS - Trait IFW

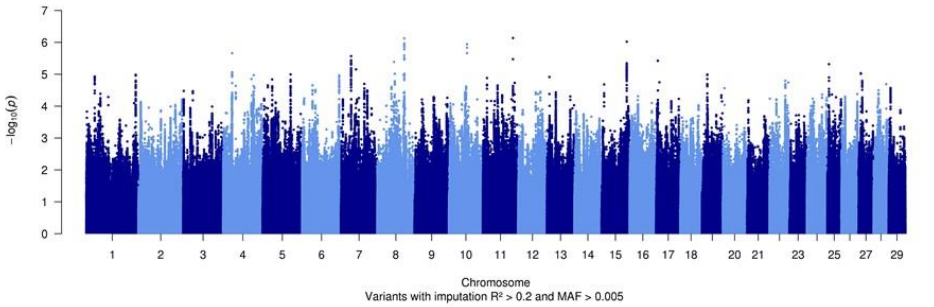

Partner INRAE - Population BLA_STEERS - Trait IFW

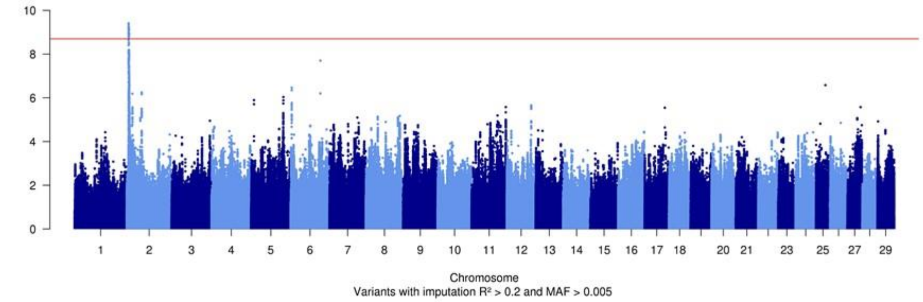

fixed effects meta-analysis method with 5 within-population GWAS

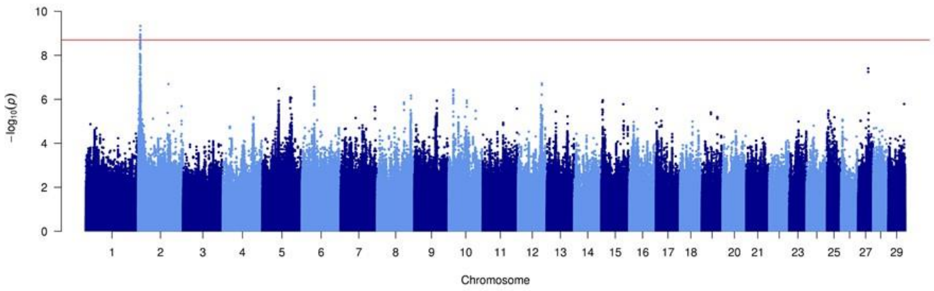

z-score meta-analysis method with 5 within-population GWAS

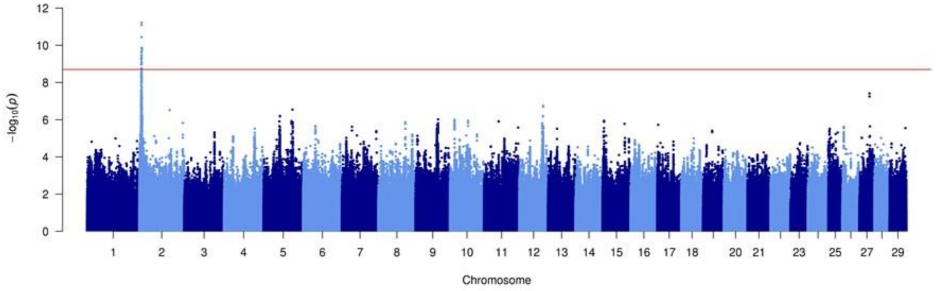

C8 meta-analysis

Partner FBN - Population HC_COWS - Trait REA

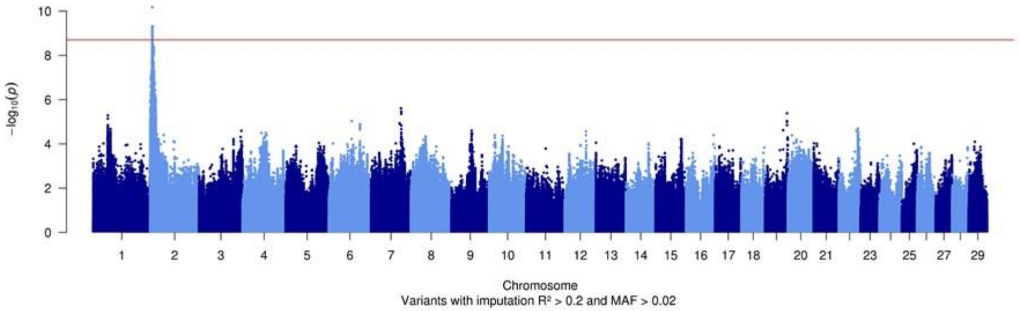

Partner UAL - Population COMP_STEERS - Trait REA

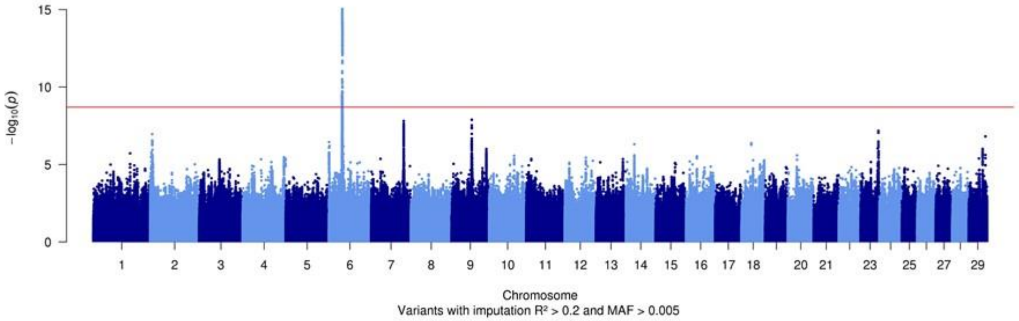

Partner FBN - Population HC_BULLS - Trait REA

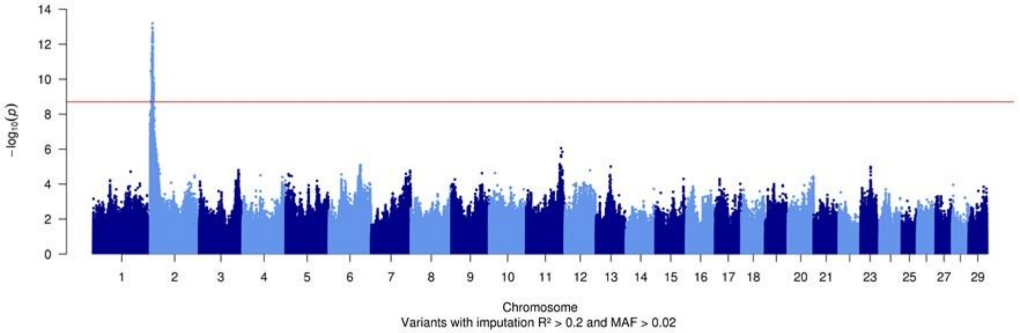

fixed effects meta-analysis method with 3 within-population GWAS

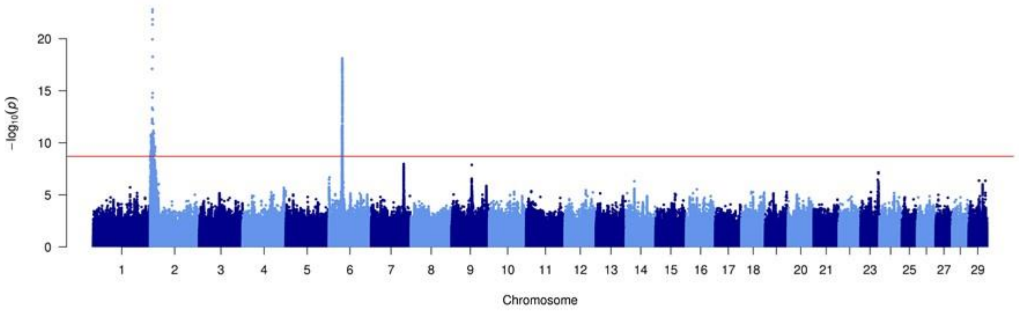

z-score meta-analysis method with 3 within-population GWAS

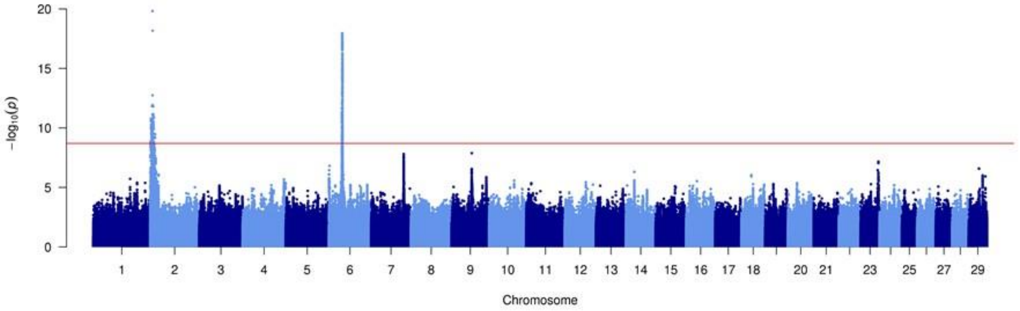

Supplement: Supplementary file 2 — Additional file 2: Figure S1. Manhattan plots of each meta-analysis from within-population GWAS and MA results. [file 12711_2023_848_MOESM2_ESM.pdf]
